# Extended Bloch–McConnell equations for mechanistic analysis of hyperpolarized ^13^C magnetic resonance experiments on enzyme systems

**DOI:** 10.5194/mr-2-421-2021

**Published:** 2021-06-15

**Authors:** Thomas R. Eykyn, Stuart J. Elliott, Philip W. Kuchel

**Affiliations:** 1 School of Biomedical Engineering and Imaging Sciences, King's College London, St Thomas' Hospital, London SE1 7EH, United Kingdom; 2 Centre de Résonance Magnétique Nucléaire à Très Hauts Champs – FRE 2034 Université de Lyon / CNRS / Université Claude Bernard Lyon 1 / ENS de Lyon, 5 Rue de la Doua, 69100 Villeurbanne, France; 3 School of Life and Environmental Sciences, University of Sydney, Sydney, NSW 2006, Australia; a current address: Department of Chemistry, University of Liverpool, Liverpool L69 7ZD, United Kingdom

## Abstract

We describe an approach to formulating the kinetic master equations of the time evolution of NMR signals in reacting (bio)chemical systems. Special focus is given to studies that employ signal enhancement (hyperpolarization) methods such as dissolution dynamic nuclear polarization (dDNP) and involving nuclear spin-bearing solutes that undergo reactions mediated by enzymes and membrane transport proteins. We extend the work given in a recent presentation on this topic (Kuchel and Shishmarev, 2020) to now include enzymes with two or more substrates and various enzyme reaction mechanisms as classified by Cleland, with particular reference to non-first-order processes. Using this approach, we can address some pressing questions in the field from a theoretical standpoint. For example, why does binding of a hyperpolarized substrate to an enzyme *not* cause an appreciable loss of the signal from the substrate or product? Why does the concentration of an unlabelled pool of substrate, for example ${}^{12}$ C lactate, cause an increase in the rate of exchange of the ${}^{13}$ C-labelled pool? To what extent is the equilibrium position of the reaction perturbed during administration of the substrate? The formalism gives a full mechanistic understanding of the time courses derived and is of relevance to ongoing clinical trials using these techniques.

## 1 Introduction

Nuclear magnetic resonance (NMR) spectroscopy and imaging (MRI) are widely employed techniques with far-reaching applications in physics, chemistry, medicine and the life sciences. NMR and MRI provide a wealth of information from structure elucidation, protein dynamics and metabolic profiling through to disease diagnostics in oncology, cardiology and neurology among others. The technique's low sensitivity is one of the primary concerns in the magnetic resonance community and is often a limiting factor in experiments from solid-state NMR to medical imaging. Recent work has shown that the sensitivity of NMR experiments can be improved by using non-equilibrium hyperpolarization techniques such as dissolution dynamic nuclear polarization (dDNP) to boost signal intensities by many orders of magnitude (Ardenkjaer-Larsen et al., 2003). Such techniques have led to new applications (Golman et al., 2003, 2006; Keshari and Wilson, 2014) and necessitated the development of acquisition strategies to exploit the hyperpolarized magnetization in a time-efficient manner (Yen et al., 2009) as well as new tools for signal processing and image reconstruction (Hu et al., 2010). A challenge with the interpretation of these recordings is that, unlike radio tracers, hyperpolarized MR is a non-tracer technique requiring the injection of physiological or even supra-physiological concentrations of substrate.

To date there have been many mathematical methods devised for analysing the kinetic time courses in dDNP NMR studies (Zierhut et al., 2010; Hill et al., 2013b; Pagès and Kuchel, 2015; Daniels et al., 2016). However, until recently there has been little consensus on the best methods for analysing and then interpreting reaction kinetics measured therein. A theoretical framework has only recently appeared to fully elucidate the underlying mechanisms (Kuchel and Shishmarev, 2020). One challenge is that the widely used Bloch–McConnell equations describe the exchange of magnetization of only the MR active nuclei, while the reaction kinetics are subject to a plethora of molecular interactions in a (bio)chemical milieu. Furthermore, in a typical hyperpolarized MR experiment the initial injection of a non-tracer concentration of substrate causes the reaction system to be perturbed from its equilibrium state, or quasi-steady state, and therefore the concentrations of the reactants are time-dependent. In this regard, challenges relate to the description of non-linear kinetics, for example second-order reactions, and the involvement of unobservable (non-labelled) metabolites to the overall kinetics, e.g. enzyme cofactors, co-substrates and natural abundance ${}^{12}$ C-containing metabolites (Hill et al., 2013a), as well as explicit descriptions of enzyme mechanisms, e.g. sequential-ordered, sequential-random, double-displacement (ping–pong) reactions, and allosteric interactions that occur on an enzyme far from its active site. Enzyme activity is also influenced by inhibitors that can be competitive, non-competitive, or uncompetitive (Cleland, 1967; Cook and Cleland, 2007). Mathematical models of enzyme systems should agree with standard descriptions of (bio)chemical kinetics while remaining capable of describing the time evolution of magnetization that is described by the Bloch–McConnell equations (McConnell, 1958).

Here we address these issues in a stepwise manner by developing a mechanistic approach that combines the MR interactions with the chemical and/or enzyme-mediated reactions described by the Bloch–McConnell equations. These equations are grounded in the concept of conservation of mass of the species responsible for the hyperpolarized signal plus its non-hyperpolarized counterpart and the various products; this was recently highlighted (Kuchel and Shishmarev, 2020) where the MR-visible signal decays to produce an MR-invisible one.

### 1.1 Basic concepts – sensitivity

We begin addressing the problem by defining the signal-to-noise ratio (SNR) in MR. In its most basic form, sensitivity is described by the ratio of the signal amplitude divided by the root mean square of the amplitude of the noise. When a signal $S\left(t\right)$ is detected in the NMR receiver coil that surrounds the sample, the magnitude of the induced current is a function of (i) the perturbation of nuclear spin populations from thermal equilibrium $S_{\mathrm{sample}}\left(t\right)$ plus (ii) a random contribution from the noise in the electronic circuitry $S_{\mathrm{electronics}}\left(t\right)$ . Hence

1 $$S\left(t\right)=S_{\mathrm{sample}}\left(t\right)+S_{\mathrm{electronics}}\left(t\right).$$

The current induced in the coil is time-dependent and proportional to the magnetization that precesses in the x,y plane. In other words, the signal $S\left(t\right)$ is recorded until decoherence renders $S_{\mathrm{sample}}\left(t\right)$ undetectable against the noise, $S_{\mathrm{electronics}}\left(t\right)$ . The latter is mainly attributed to the radiofrequency (RF) circuitry in the probe head and the preamplifier(s) (e.g. Johnson noise – Johnson, 1928) of the spectrometer. If the NMR signal (free induction decay; FID) that is detected in a subsequent experiment is indistinguishable from the first and the two are added together, then the signal amplitude (peak area) will scale linearly with the number of added FIDs, N . The noise associated with each experiment is random, and assuming its source remains fixed over time, i.e. stationary noise, then the amplitude scales with the square root of the number of FIDs, $N^{1/2}$ . Hence signal summation enhances the SNR of an NMR experiment in proportion to the square root of the number of FIDs. In other words, to achieve an enhancement by a factor ξ requires an increase in experiment duration of $\xi^{2}$ . Therefore, unavoidably, FID summation is a slow process, and experiments can sometimes take days or weeks to achieve a sufficient SNR from a sample of a low-sensitivity nuclide or one with a long relaxation time. The amount of attainable signal averaging is constrained when monitoring dynamic processes by NMR spectroscopy, and an inherently good SNR is required from the outset for a time course experiment.

### 1.2 Thermal effects

The usual way to proceed when calculating the NMR response of a spin system to RF pulse sequences is to solve the ordinary quantum mechanical master equation that describes the evolution of the spin density operator (Hore et al., 2015). This is the Liouville–von Neumann equation, which has been extended to include non-coherent interactions (predominantly relaxation phenomena) (Ernst et al., 1987):

2 $$\frac{d}{dt}\rho =-i\hat{H}\rho -\hat{\Gamma }\left(\rho -\rho_{0}\right),$$

where $\hat{H}$ is the commutation superoperator of the coherent Hamiltonian H given by $\hat{H}\rho =\left[H,\rho \right]$ , which contains information on all spin–spin and field–spin interactions, while $\hat{\Gamma }$ is the relaxation superoperator that describes all longitudinal ( $T_{1}$ ) and transverse ( $T_{2}$ ) relaxation processes, as well as any cross-relaxation or cross-correlation interactions. Note that in the interests of reducing clutter in equations (for which the operator context should be clear), hereafter we have omitted carets denoting operators and only used them to denote superoperators.

Our aim here is to describe the kinetics of exchange between different solutes that contain hyperpolarized nuclei, e.g. A↔B , in which the relaxation times are constant. In this quest, the first simplifying assumption that is worth exploring is that all intermolecular interactions, notably scalar coupling, dipolar coupling, cross-relaxation and cross-correlation between species A and B , can be ignored. This applies to non-interacting solute molecules in solution in which motional averaging occurs, and we focus on thermal effects on the evolution of the FID.

The so-called Zeeman polarization term describes the sensitivity of $S_{\mathrm{sample}}\left(t\right)$ in Eq. (1) to temperature and magnetic field in an NMR experiment. Magnetic polarization is described by the equilibrium density operator $\rho_{0}$ that specifies the probability distribution of states. Zeeman polarization corresponds to the magnitude of normalized longitudinal spin order $I_{z}$ that is contained in $\rho_{0}$ . Specifically, for an ensemble of spin- $\frac{1}{2}$ nuclei this is given by (Ernst et al., 1987)

3 $$\rho_{0}=\frac{\mathrm{exp}(-\hbar H_{0}/kT)}{\mathrm{Tr}\{\mathrm{exp}(-\hbar H_{0}/kT)\}},$$

where k is the Boltzmann constant and T is the temperature (Kelvin). The Zeeman Hamiltonian $H_{0}$ describes the interaction of the spins with the static magnetic field of magnitude $B_{0}$ , given by $H_{0}=\omega_{0}I_{z}$ , where $\omega_{0}$ is the Larmor frequency (rad s ${}^{-1}$ ). In the basis of the two eigenstates $|\alpha \rangle$ (“spin-up”) and $|\beta \rangle$ (“spin-down”), the equilibrium density operator is written in matrix form as

4 $$\rho_{0}=\frac{1}{Z}\left[\begin{array}{cc} \mathrm{exp}(\hbar \omega_{0}/2kT) & 0\\ 0 & \mathrm{exp}(-\hbar \omega_{0}/2kT) \end{array}\right],$$

where Z is the partition function, given by $Z=\sum_{i=1}^{M}\mathrm{exp}\left(-\varepsilon_{i}/kT\right)$ , and M is the number of states ( M=2 for an $I=\frac{1}{2}$ nucleus). In the case of a spin- $\frac{1}{2}$ system, the partition function is the sum of the populations $Z=\mathrm{exp}(\hbar \omega_{0}/2kT)+\mathrm{exp}(-\hbar \omega_{0}/2kT)\approx 2$ when $\varepsilon_{i}$ is very small, as is typically the case at thermal equilibrium in NMR systems. The Zeeman polarization is proportional to the projection of the spin density operator onto the angular momentum operator. In other words, it is proportional to the expectation value of $\langle I_{z}\rangle$ and is given by (Keeler, 2010)

5 $$\langle I_{z}\rangle =\mathrm{Tr}[\rho_{0}I_{z}]=\frac{1}{2Z}[\mathrm{exp}(\hbar \omega_{0}/2kT)-\mathrm{exp}(-\hbar \omega_{0}/2kT)].$$

Hence, the Zeeman polarization for an ensemble of nuclear spins is the normalized *imbalance* between the populations of the $|\alpha \rangle$ and $|\beta \rangle$ states, $p_{\alpha }$ and $p_{\beta }$ , respectively; in other words, it is the normalized net population difference that is given by

6 $$P=\frac{p_{\alpha }-p_{\beta }}{p_{\alpha }+p_{\beta }}.$$

This normalization is carried out with respect to the total population of the nuclear ensemble such that $p_{\alpha }+p_{\beta }=1$ . Therefore, the bounds on the polarization are -1<P<+1 . At room temperature ( ∼298 K) and in a field of 11.75 T (500 MHz for ${}^{1}$ H nuclei), the thermal equilibrium Zeeman polarization, $P_{z,\mathrm{eq}}$ , is a mere $∼4\times 10^{-5}$ . Thus, there is only a tiny population difference between the spin states of a nuclear ensemble, which implies inherently weak polarization. It is this small population imbalance which is manipulated in NMR experiments under thermal equilibrium conditions. This weak polarization is a consequence of the small difference in energy ( ∼0.1 J mol ${}^{-1}$ ) between nuclear spin energy levels at room temperature ( ∼2.5 kJ mol ${}^{-1}$ ), and it implies only weak alignment of nuclear spins in the static magnetic field of all contemporary superconducting magnets.

In the usual quantum mechanical analysis of multiple spin systems, the density operator (that describes the probability density of states) is normalized to 1, meaning that the summed (total) probability density of all states is 1. This is expressed mathematically as $\mathrm{Tr}\left[\rho \right]=1$ , where Tr denotes the trace of the matrix (Hore et al., 2015). To describe non-equilibrium reactions in terms of solute concentrations requires a scaled density operator (Kuhne et al., 1979):

7 $$\sigma_{i}=\left[A_{i}\right]\rho_{i},$$

where $\sigma_{i}$ is now proportional to concentration [ $A_{i}$ ]. Differentiation of Eq. (7) leads to

8 $$\frac{d\sigma_{i}}{dt}=\left[A_{i}\right]\frac{d\rho_{i}}{dt}+\frac{d\left[A_{i}\right]}{dt}\rho_{i}.$$

Therefore, it follows that for a system at chemical equilibrium $d\left[A_{i}\right]/dt=0$ , the scaled density operator is directly proportional to the normalized density operator. For non-equilibrium systems the concentrations are time-dependent viz. $d\left[A_{i}\right]/dt\neq 0$ , so the two no longer scale in a straightforward manner.

On the other hand, equilibrium magnetization ( $M_{z,\mathrm{eq}}$ ) is a bulk property that is the net magnetic dipole moment per unit volume and is proportional to $\langle I_{z}\rangle$ , where the proportionality factor is Nℏγ . From Eq. (5) this yields the expression for the magnetization in terms of magnetic field strength, temperature and number of spins in the sample (or more specifically in the detection volume of the NMR spectrometer):

9 $$M_{z,\mathrm{eq}}=\frac{N\hbar \gamma }{2}\mathrm{tanh}\left(\frac{\hbar \gamma B_{0}}{2kT}\right).$$

In the so-called “high-temperature limit” (room temperature, in the cases addressed here), Eq. (9) simplifies to

10 $$M_{z,\mathrm{eq}}=\frac{N\hbar^{2}\gamma^{2}B_{0}}{4kT}.$$

In words, “thermal magnetization” is proportional to the magnitude of the external magnetic field strength, $B_{0}$ , and is inversely proportional to the temperature, T , while being proportional to the number of spins, N . Therefore, it is *proportional* to the concentration [ $A_{i}$ ] of the solute that bears the NMR-active nucleus.

## 2 Equation of motion – the Bloch equations

In the absence of intermolecular binding (however transient) or scalar couplings, the motion (time evolution) of magnetizations is described by the Bloch equations. Magnetization is explicitly declared to be proportional to reactant concentrations [ A ] and [ B ], as has recently been discussed (Kuchel and Shishmarev, 2020). To explore this situation, we start with the basic Bloch equations for a single spin- $\frac{1}{2}$ ensemble. The equation describes the time evolution of x,y and z magnetization in the rotating frame and includes the influence of chemical shift, RF fields, and transverse ( $T_{2}$ ) and longitudinal relaxation ( $T_{1}$ ) time constants. The Bloch equations in their complete form are described as being inhomogeneous, and they can be written using a matrix and vectors:

11 $$\begin{array}{rl} \frac{d}{dt}\left[\begin{array}{c} M_{x}\\ M_{y}\\ M_{z} \end{array}\right] & =-\left[\begin{array}{ccc} R_{2} & \Omega & -\omega_{y}\\ -\Omega & R_{2} & \omega_{x}\\ \omega_{y} & -\omega_{x} & R_{1} \end{array}\right]\left[\begin{array}{c} M_{x}\\ M_{y}\\ M_{z} \end{array}\right]\\ & +\left[\begin{array}{c} 0\\ 0\\ R_{1}M_{z,\mathrm{eq}} \end{array}\right], \end{array}$$

where $\Omega =\omega_{0}-\omega_{\mathrm{RF}}$ is the “offset frequency” in the rotating frame $;\;\omega_{0}$ (rad s ${}^{-1}$ ) is the Larmor frequency; $\omega_{\mathrm{RF}}$ (rad s ${}^{-1}$ ) is the RF frequency; the x component of the RF field (rad s ${}^{-1}$ ) is $\omega_{x}=-\gamma B_{1}\mathrm{cos}(\omega_{\mathrm{RF}}t+\varphi$ ); and the y component is $\omega_{y}=-\gamma B_{1}\mathrm{sin}(\omega_{\mathrm{RF}}t+\varphi$ ), where the magnitude of the field strength is $B_{1}$ and the phase of the wave form relative to an internal reference source is φ . The longitudinal relaxation rate constant is denoted by $R_{1}=1/T_{1}$ , the transverse one by $R_{2}=1/T_{2}$ , and the equilibrium magnetization by $M_{z,\mathrm{eq}}$ .

Equation (11) is tedious to solve analytically, but it is readily solved numerically (Allard et al., 1998; Helgstrand et al., 2000). On the other hand, by including the identity operator in the basis set and adding a constant to the equilibrium magnetization (Levitt and Dibari, 1992), we obtain a much more compliant (to analysis) matrix equation:

12 $$\frac{d}{dt}\left[\begin{array}{c} \frac{E}{2}\\ M_{x}\\ M_{y}\\ M_{z} \end{array}\right]=-\left[\begin{array}{cccc} 0 & 0 & 0 & 0\\ 0 & R_{2} & \Omega & -\omega_{y}\\ 0 & -\Omega & R_{2} & \omega_{x}\\ -2\Theta & \omega_{y} & -\omega_{x} & R_{1} \end{array}\right]\left[\begin{array}{c} \frac{E}{2}\\ M_{x}\\ M_{y}\\ M_{z} \end{array}\right],$$

where E is equal to 1 and the factor $\Theta =R_{1}M_{z,\mathrm{eq}}$ describes the equilibrium magnetization.

### 2.1 Chemical exchange kinetics of systems prior to and at equilibrium – the Bloch–McConnell equations

We can extend the system of equations from describing an ensemble of single spins to two or more exchanging spins. The system of equations now accounts for the magnetization interaction with the lattice and exchange via the forward and reverse chemical reactions. These are the Bloch–McConnell equations (McConnell, 1958).

First, consider the rate expressions for a simple bi-directional chemical reaction. The coupled differential equations describing first-order reaction kinetics of solute A becoming solute B and back again, A↔B , are typically expressed in terms of molar concentrations:

13dAtdt=-k1At+k-1Bt,14dBtdt=k1At-k-1Bt,

which can be expressed in matrix form:

15 $$\frac{d}{dt}\left[\begin{array}{c} \left[A\left(t\right)\right]\\ \left[B\left(t\right)\right] \end{array}\right]=\left[\begin{array}{cc} -k_{1} & k_{-1}\\ k_{1} & -k_{-1} \end{array}\right]\left[\begin{array}{c} \left[A\left(t\right)\right]\\ \left[B\left(t\right)\right] \end{array}\right].$$

The rate constant for the forward reaction is denoted by $k_{1}$ , while for the reverse reaction it is $k_{-1}$ . The time-dependent concentrations are given by $\left[A\left(t\right)\right]$ and $\left[B\left(t\right)\right]$ . As required by the fact that this is a closed system, the equations must conform to the *principle of conservation of mass*. Specifically, the sum of the rates of change in $\left[A\left(t\right)\right]$ and $\left[B\left(t\right)\right]$ given by $d\left[A\left(t\right)\right]/dt+d\left[B\left(t\right)\right]/dt$ is zero. We return to this point below. In other words, mass is neither created nor destroyed during the reaction in such a closed system.

For the simplest case of two magnetically active solutes, each possessing a single spin- $\frac{1}{2}$ nuclide, in chemical exchange, A↔B , the direct product (a mathematical operation used in quantum mechanics to generate the necessary combinations of states) of the chemical (solute) space $\left\{\left[A\right],\left[B\right]\right\}$ and the magnetization vector space $\left\{M_{x},M_{y},M_{z}\right\}$ for each of A and B is given by

16 $$\left[\begin{array}{c} 1\\ 1 \end{array}\right]⊗\left[\begin{array}{c} M_{x}\\ M_{y}\\ M_{z} \end{array}\right]=\left[\begin{array}{c} M_{x}^{A}\\ M_{y}^{A}\\ M_{z}^{A}\\ M_{x}^{B}\\ M_{y}^{B}\\ M_{z}^{B} \end{array}\right].$$

A new exchange matrix in the basis of the new magnetization space $\left\{M_{x}^{A},M_{y}^{A},M_{z}^{A},M_{x}^{B},M_{y}^{B},M_{z}^{B}\right\}$ is calculated by taking the direct product of the exchange matrix with the identity operator I that is chosen to have the same dimensions as the magnetization space. The direct product is given by

17 $$\begin{array}{rl} & \left[\begin{array}{cc} -k_{1} & k_{-1}\\ k_{1} & -k_{-1} \end{array}\right]⊗\left[\begin{array}{ccc} 1 & 0 & 0\\ 0 & 1 & 0\\ 0 & 0 & 1 \end{array}\right]=\\ & \left[\begin{array}{cccccc} -k_{1} & 0 & 0 & k_{-1} & 0 & 0\\ 0 & -k_{1} & 0 & 0 & k_{-1} & 0\\ 0 & 0 & -k_{1} & 0 & 0 & k_{-1}\\ k_{1} & 0 & 0 & -k_{-1} & 0 & 0\\ 0 & k_{1} & 0 & 0 & -k_{-1} & 0\\ 0 & 0 & k_{1} & 0 & 0 & -k_{-1} \end{array}\right]. \end{array}$$

Likewise, the matrix describing coherent and incoherent magnetization interactions can be recast in a similar fashion to give

18 $$\begin{array}{rl} & \left[\begin{array}{cc} 1 & 0\\ 0 & 1 \end{array}\right]⊗\left[\begin{array}{ccc} R_{2} & \Omega & -\omega_{y}\\ -\Omega & R_{2} & \omega_{x}\\ \omega_{y} & -\omega_{x} & R_{1} \end{array}\right]\\ & =\left[\begin{array}{cccccc} R_{2}^{A} & \Omega^{A} & -\omega_{y} & 0 & 0 & 0\\ -\Omega^{A} & R_{2}^{A} & \omega_{x} & 0 & 0 & 0\\ \omega_{y} & -\omega_{x} & R_{1}^{A} & 0 & 0 & 0\\ 0 & 0 & 0 & R_{2}^{B} & \Omega^{B} & -\omega_{y}\\ 0 & 0 & 0 & -\Omega^{B} & R_{2}^{B} & \omega_{x}\\ 0 & 0 & 0 & \omega_{y} & -\omega_{x} & R_{1}^{B} \end{array}\right]. \end{array}$$

The inhomogeneous form of the Bloch equations can now be constructed to take into account both the coherent and incoherent interactions *as well as* chemical exchange. This yields the inhomogeneous form of the Bloch–McConnell equations, which are written (again in matrix form) as

19 $$\begin{array}{rl} & \frac{d}{dt}\left[\begin{array}{c} M_{x}^{A}\\ M_{y}^{A}\\ M_{z}^{A}\\ M_{x}^{B}\\ M_{y}^{B}\\ M_{z}^{B} \end{array}\right]=\\ & -\left[\begin{array}{cccccc} R_{2}^{A}+k_{1} & \Omega^{A} & -\omega_{y} & -k_{-1} & 0 & 0\\ -\Omega^{A} & R_{2}^{A}+k_{1} & \omega_{x} & 0 & -k_{-1} & 0\\ \omega_{y} & -\omega_{x} & R_{1}^{A}+k_{1} & 0 & 0 & -k_{-1}\\ -k_{1} & 0 & 0 & R_{2}^{B}+k_{-1} & \Omega^{B} & -\omega_{y}\\ 0 & -k_{1} & 0 & -\Omega^{B} & R_{2}^{B}+k_{-1} & \omega_{x}\\ 0 & 0 & -k_{1} & \omega_{y} & -\omega_{x} & R_{1}^{B}+k_{-1} \end{array}\right]\\ & \left[\begin{array}{c} M_{x}^{A}\\ M_{y}^{A}\\ M_{z}^{A}\\ M_{x}^{B}\\ M_{y}^{B}\\ M_{z}^{B} \end{array}\right]+\left[\begin{array}{c} 0\\ 0\\ R_{1}^{A}M_{z,\mathrm{eq}}^{A}\\ 0\\ 0\\ R_{1}^{B}M_{z,\mathrm{eq}}^{B} \end{array}\right], \end{array}$$

where $M_{z,\mathrm{eq}}^{A}$ and $M_{z,\mathrm{eq}}^{B}$ denote the respective equilibrium magnetizations (hence the subscript eq).

The inhomogeneous form of the Bloch–McConnell equations can similarly be modified by incorporating the equilibrium magnetization to create a homogeneous form of this master equation:

20 $$\begin{array}{rl} & \frac{d}{dt}\left[\begin{array}{c} \frac{E}{2}\\ M_{x}^{A}\\ M_{y}^{A}\\ M_{z}^{A}\\ M_{x}^{B}\\ M_{y}^{B}\\ M_{z}^{B} \end{array}\right]=\\ & -\left[\begin{array}{ccccccc} 0 & 0 & 0 & 0 & 0 & 0 & 0\\ 0 & R_{2}^{A}+k_{1} & \Omega^{A} & -\omega_{y} & -k_{-1} & 0 & 0\\ 0 & -\Omega^{A} & R_{2}^{A}+k_{1} & \omega_{x} & 0 & -k_{-1} & 0\\ -2\Theta^{A} & \omega_{y} & -\omega_{x} & R_{1}^{A}+k_{1} & 0 & 0 & -k_{-1}\\ 0 & -k_{1} & 0 & 0 & R_{2}^{B}+k_{-1} & \Omega^{B} & -\omega_{y}\\ 0 & 0 & -k_{1} & 0 & -\Omega^{B} & R_{2}^{B}+k_{-1} & \omega_{x}\\ -2\Theta^{B} & 0 & 0 & -k_{1} & \omega_{y} & -\omega_{x} & R_{1}^{B}+k_{-1} \end{array}\right]\\ & \left[\begin{array}{c} \frac{E}{2}\\ M_{x}^{A}\\ M_{y}^{A}\\ M_{z}^{A}\\ M_{x}^{B}\\ M_{y}^{B}\\ M_{z}^{B} \end{array}\right]. \end{array}$$

Again, the factors $\Theta^{A}=R_{1}^{A}M_{z,\mathrm{eq}}^{A}$ and $\Theta^{B}=R_{1}^{B}M_{z,\mathrm{eq}}^{B}$ account for the respective equilibrium magnetizations.

#### 2.1.1 Simulations of thermal kinetics using Eq. (19)

**Figure 1:**
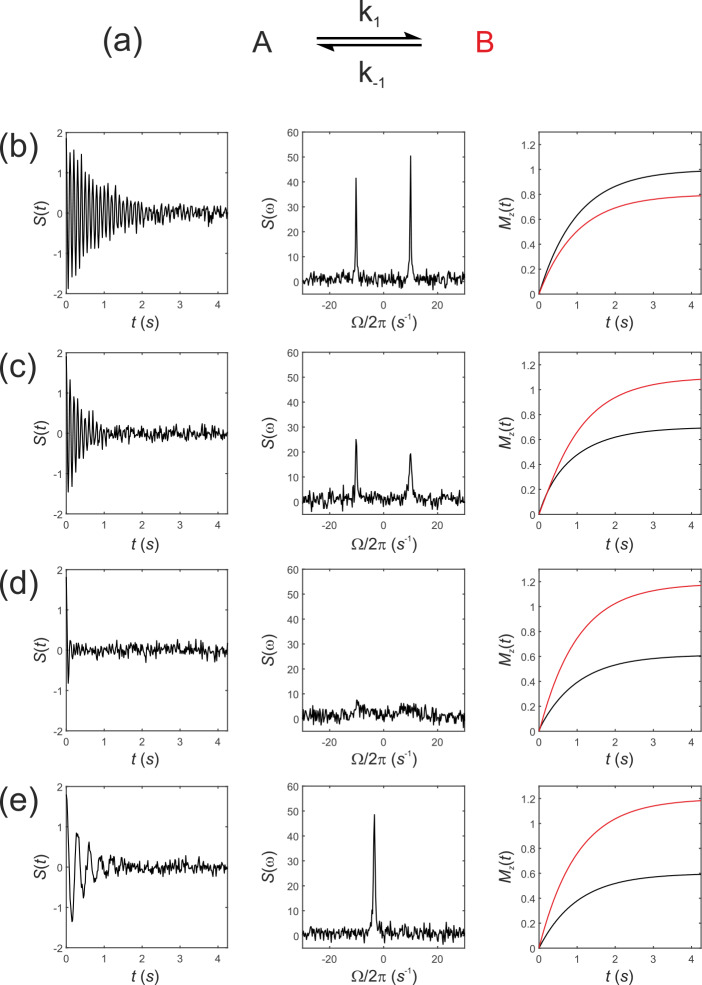
Simulated NMR spectra resulting from a two-site exchange process between *thermally polarized* solutes, A↔B , shown schematically in **(a)**. Simulated FIDs $S\left(t\right)$ are shown in **(b)**–**(e)**, left panel, with corresponding spectra $s\left(\omega \right)$ , middle panel, and the recovery of z magnetizations, $M_{z}^{A}\left(t\right)$ and $M_{z}^{B}\left(t\right)$ , right panel. Spectra were simulated with rate constants, **(b)** $k_{1}=k_{-1}=0$ ; **(c)** $k_{1}=2$ s ${}^{-1}$ , $k_{-1}=1$ s ${}^{-1}$ ; **(d)** $k_{1}=20$ s ${}^{-1}$ , $k_{-1}=10$ s ${}^{-1}$ ; and **(e)** $k_{1}=2000$ s ${}^{-1}$ , $k_{-1}=1000$ s ${}^{-1}$ , corresponding to no exchange, slow, intermediate, and fast exchange regimes, respectively.

Next, consider Eq. (19) for simulating the evolution of the x,y , and z components of the magnetization of a “thermal magnetization” (*non-hyperpolarized*) sample. We seek the NMR spectrum that results from a two-site exchange reaction between solutes A and B , Fig. 1a, as conventionally observed in room temperature NMR experiments.

Simulations were performed in *MatLab* with equilibrium z magnetizations $M_{z,\mathrm{eq}}^{A}=1.0$ and $M_{z,\mathrm{eq}}^{B}=0.8$ and an initial magnetization vector given by $M_{0}=\left[0,0,1.0,0,0,0.8\right]$ . Chemical shift offsets were $\Omega^{A}=10\times 2\pi$ rad s ${}^{-1}$ and $\Omega^{B}=-10\times 2\pi$ rad s ${}^{-1}$ . Relaxation rate constants were $R_{1}^{A}=R_{1}^{B}=1$ s ${}^{-1}$ and $R_{2}^{A}=R_{2}^{B}=1$ s ${}^{-1}$ . The influence of an RF ${}_{y}$ pulse was then calculated with $\omega_{x}=-\gamma B_{1}\mathrm{cos}\left(\pi /2\right)$ and $\omega_{y}=-\gamma B_{1}\mathrm{sin}\left(\pi /2\right)$ and with a field strength of 1.5 kHz, corresponding to $\omega_{y}=-\gamma B_{1}=-1500\times 2\pi$ rad s ${}^{-1}$ and $\omega_{x}=0$ . For a 90 ${}^{\circ }$ RF nutation (flip) angle the pulse duration is $t_{p}=\pi /2\omega_{y}$ , which gave a transformed magnetization vector after the pulse of $M\left(t\right)=\left[0.999,0.007,0.000,0.800,-0.005,0.000\right]$ ; this was composed mostly of $M_{x}^{A}+M_{x}^{B}$ with a residual contribution from $M_{y}^{A}+M_{y}^{B}$ arising from evolution of the chemical shift during the RF pulse and a small contribution from $M_{z}^{A}+M_{z}^{B}$ due to return of the magnetization to the equilibrium state.

The observable signal (the FID, which is a function of time) is proportional to the complex signal $S\left(t\right)=M_{x}^{A}\left(t\right)-iM_{y}^{A}\left(t\right)+M_{x}^{B}\left(t\right)-iM_{y}^{B}\left(t\right)$ . Noise was simulated by adding to the FID a normally distributed complex random vector with mean = 0 and standard deviation (SD) = 0.1. The spectrum $s\left(\omega \right)$ was then calculated by taking the Fourier transform of $S\left(t\right)$ . Simulated FIDs, $S\left(t\right)$ , are shown in Fig. 1b–e left panel, the corresponding spectra $s\left(\omega \right)$ in Fig. 1b–e middle panel, and the recovery of the z magnetizations $M_{z}^{A}\left(t\right)$ and $M_{z}^{B}\left(t\right)$ are shown in Fig. 1b–e, right panel. Spectra were simulated for a range of rate constants, where exchange was either absent, $k_{1}=k_{-1}=0$ , Fig. 1b, or for increasing rates of exchange. Thus, (c) $k_{1}=2$ s ${}^{-1}$ , $k_{-1}=1$ s ${}^{-1}$ ; (d) $k_{1}=20$ s ${}^{-1}$ , $k_{-1}=10$ s ${}^{-1}$ ; and (e) $k_{1}=2000$ s ${}^{-1}$ , $k_{-1}=1000$ s ${}^{-1}$ , corresponding to the slow, intermediate and fast regimes, respectively.

The equilibrium constant was fixed so that $K=k_{1}/k_{-1}=2$ ; hence the system was not at chemical equilibrium at t=0 s. The simulations highlight an important point: in the absence of exchange the Bloch–McConnell equations predict the recovery of the z magnetizations back to their magnetic equilibrium values $M_{z,\mathrm{eq}}^{A}$ and $M_{z,\mathrm{eq}}^{B}$ , while under conditions of fast exchange this no longer takes place during the experiment. A non-equilibrium system will rapidly recover to its chemical equilibrium but not to its initial thermal equilibrium $M_{z,\mathrm{eq}}^{A}$ and $M_{z,\mathrm{eq}}^{B}$ ; again, in other words, this does not take place within the timescale of the NMR experiment, which is typically within five $T_{1}$ values.

### 2.2 Describing hyperpolarized kinetics with the Bloch–McConnell equations

We now consider the predictions made by using Eq. (19) when simulating the evolution of the x , y , and z components of the magnetization of a hyperpolarized sample and the resulting spectrum for a two-site exchange reaction between solutes A and B . In the previous example the initial condition was $M_{z}^{A}\left(0\right)=1.0$ and $M_{z}^{B}\left(0\right)=0.8$ . To extend the Bloch–McConnell formalism to be able to predict the dynamics of a hyperpolarized experiment, we recognize that for the same magnitude of noise in the receiver circuit (although this may not be true for a hyperpolarized sample) the initial hyperpolarized magnetization is given by

21 $$M_{z,\mathrm{hyp}}=\eta M_{z,\mathrm{eq}},$$

where η is the enhancement factor that varies from one hyperpolarization experiment to another. In the case of dDNP experiments $\eta ≅10^{4}$ is typical, although this depends on the method of hyperpolarization, the solute(s) in question and a set of physicochemical parameters that are described in detail in e.g. Ardenkjaer-Larsen et al. (2015).

#### Simulations of hyperpolarized kinetics using Eq. (19)

**Figure 2:**
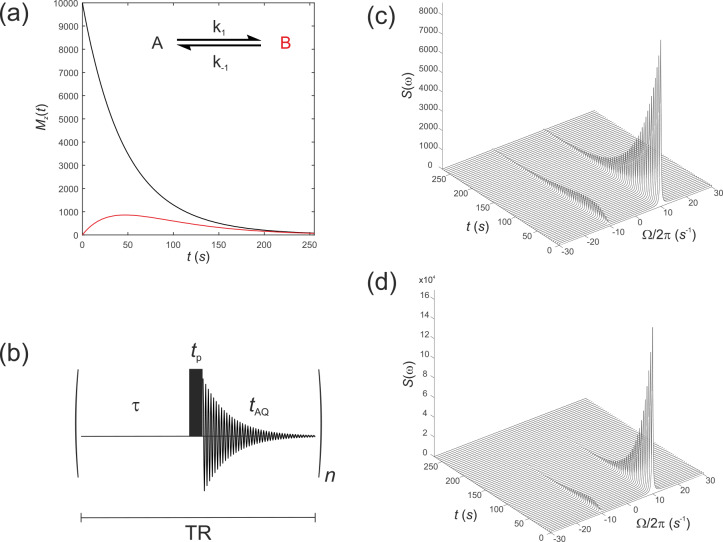
**(a)** Simulated evolution of the z components of the magnetization $M_{z}^{A}$ and $M_{z}^{B}$ for a *hyperpolarized* solute $M_{z}^{A}\left(0\right)=1\times 10^{4}$ undergoing a two-site exchange reaction, A↔B . Longitudinal relaxation rate constants were $R_{1}^{A}=R_{1}^{B}=1/60$ s ${}^{-1}$ and $R_{2}^{A}=R_{2}^{B}=1$ s ${}^{-1}$ . Rate constants were $k_{1}=k_{-1}=0.005$ s ${}^{-1}$ . **(b)** Simple pulse sequence for acquiring a time course experiment with multiple sampling of the magnetization and acquisition of an FID at each time point. **(c–d)** Waterfall plots of simulated spectra resulting from sequential application of the pulse sequence in **(b)** for an initial hyperpolarized solute A undergoing two-site exchange with solute B , calculated with a flip angle: **(c)** β=1 ${}^{\circ }$ ; and **(d)** β=20 ${}^{\circ }$ .

These were performed with equilibrium z magnetizations $M_{z,\mathrm{eq}}^{A}=1.0$ and $M_{z,\mathrm{eq}}^{B}=0.8$ , as used above, but now with an initial magnetization vector $M\left(0\right)=\left[0,0,1.0\times 10^{4},0,0,0\right]$ . This situation corresponds to an initial hyperpolarized magnetization $M_{z,\mathrm{hyp}}^{A}\left(0\right)$ of only solute A and an enhancement factor of $\eta =10^{4}$ . Chemical shifts were $\Omega^{A}=10\times 2\pi$ rad s ${}^{-1}$ and $\Omega^{B}=-10\times 2\pi$ rad s ${}^{-1}$ , while relaxation times were increased to represent a hyperpolarized ${}^{13}$ C substrate, $R_{1}^{A}=R_{1}^{B}=1/60$ s ${}^{-1}$ and $R_{2}^{A}=R_{2}^{B}=1$ s ${}^{-1}$ , with the rate constants representing an enzyme-mediated cell reaction $k_{1}=k_{-1}=0.005$ s ${}^{-1}$ . Figure 2a shows the time evolution of the z components of the magnetization, displaying the familiar (Day et al., 2007) bi-exponential time dependence of $M_{z,\mathrm{hyp}}^{A}\left(t\right)$ and $M_{z,\mathrm{hyp}}^{B}\left(t\right)$ magnetizations.

We next simulate the effect of applying the pulse sequence shown in Fig. 2b corresponding to a time course type of experiment with multiple sampling of the magnetization and acquisition of an FID at each time point. This is representative of real experiments that have been presented in the literature (Gabellieri et al., 2008; Hill et al., 2013b). The time delays correspond to a pre-scan delay τ , the duration of the pulse $t_{p}$ and the duration of the FID $t_{\mathrm{aq}}$ . The experiment is repeated n times to sample the entire time course where the temporal resolution is then given by the total repetition time $\mathrm{TR}=\tau +t_{p}+t_{\mathrm{aq}}$ , and the total duration of the experiment is given by nTR . In this experiment we make the assumption that the transverse magnetization from one experiment to the next is not recovered by the application of a subsequent pulse. This assumption is reasonable provided the acquisition time is much longer that the time taken for the FID to decay to zero, namely $t_{\mathrm{aq}}\gg T_{2}^{*}$ .

The influence of this pulse sequence was then calculated, accounting for multiple sampling of the magnetization. The RF pulse was again specified by $\omega_{x}=-\gamma B_{1}\mathrm{cos}\left(\pi /2\right)$ and $\omega_{y}=-\gamma B_{1}\mathrm{sin}\left(\pi /2\right)$ with a field strength of 1.5 kHz, which corresponds to $\omega_{y}=-\gamma B_{1}=-1500\times 2\pi$ rad s ${}^{-1}$ . Application of an RF pulse tilts the hyperpolarized magnetization away from the z axis by an angle of β radians. The magnitude of the observable transverse magnetization is proportional to sin( β ), and the remaining longitudinal magnetization is proportional to cos( β ).

Simulations were performed with the same magnitude of noise as in Fig. 1. The time evolution of the magnetization was recorded for the pulse sequence shown in Fig. 2b with sequential acquisition of 64 spectra and a repetition time of TR=4.25 s. The effect of acquiring a time series of spectra with either a flip angle β=1 ${}^{\circ }$ , Fig. 2c, or β=20 ${}^{\circ }$ , Fig. 2d, are seen in the stack plots. The pulse length (duration) was $t_{p}=\beta \pi /180\omega_{y}$ . After a single β=1 ${}^{\circ }$ pulse was applied to $M\left(0\right)$ , the magnetization vector was tilted to become $M\left(t\right)=\left[0.174,0.000,9.998,0.000,0.000,0.000\right]\times 10^{3}$ prior to acquisition of the FID. This was composed mostly of $M_{z}^{A}$ with a small contribution from $M_{x}^{A}$ that arose from excitation by the β=1 ${}^{\circ }$ pulse or, following a β=20 ${}^{\circ }$ pulse the magnetization vector was tilted to become $M\left(t\right)=\left[3.420,0.004,9.397,0.000,0.000,0.000\right]\times 10^{3}$ , again composed mostly of $M_{z}^{A}$ but with a greater contribution from $M_{x}^{A}$ due to excitation by a pulse with a larger value of β . Since the magnetization relaxed to its thermal equilibrium state, the hyperpolarized magnetization was effectively destroyed during application of the RF (sampling) pulse, and it was not re-generated. This may not be the outcome when non-linear effects such as radiation damping cause recovery of the hyperpolarized signal (Weber et al., 2019).

The z magnetization after the application of a single RF pulse and delay TR is therefore given by

22 $$S(\mathrm{TR})=S(0)\mathrm{cos}(\theta )\mathrm{exp}\left(-R_{1}\mathrm{TR}\right).$$

Following the application of a series of n RF pulses with a total delay nTR=t , the signal is given by (Kuchel and Shishmarev, 2020)

23 $$S(t)=S(0){\mathrm{cos}}^{n}(\theta )\mathrm{exp}\left(-R_{1}t\right).$$

The apparent relaxation time constant of the hyperpolarized signal, including the influence of both the intrinsic $T_{1}$ and flip angle correction, is given by Hill et al. (2013b) and Kuchel and Shishmarev (2020):

24exp⁡-R1,appt=cos⁡n(θ)exp⁡-R1t,25R1,app=R1-1TRln⁡cos⁡(θ).

In the previous examples in Fig. 2c and d, with a typical $T_{1}=60$ s (Keshari and Wilson, 2014) corresponding to $R_{1}=1.67\times 10^{-2}$ s ${}^{-1}$ and a TR=4.25 s, the flip angle correction for a β=1 ${}^{\circ }$ pulse was $3.58\times 10^{-5}$ , which “for all intents and purposes” is negligible, giving $R_{1,\mathrm{app}}=1.67\times 10^{-2}$ s ${}^{-1}$ and $T_{1,\mathrm{app}}=59.87$ s. Hence, the time dependence of the signal shown in Fig. 2c is a robust reflection of the $M_{z}\left(t\right)$ seen in Fig. 2a. For β=20 ${}^{\circ }$ the flip angle correction was $1.46\times 10^{-2}$ , giving $R_{1,\mathrm{app}}=3.13\times 10^{-2}$ s ${}^{-1}$ and $T_{1,\mathrm{app}}=31.95$ s. Therefore, for the larger flip angle there was a tradeoff between the increased sensitivity and the corresponding reduction in $T_{1,\mathrm{app}}$ with the more rapid decay of the NMR signal. The time dependence seen in Fig. 2d is no longer a good reflection of the $M_{z}\left(t\right)$ shown in Fig. 2a. We conclude that when the RF flip angle is small, <1 ${}^{\circ }$ , and the magnetization is sampled many times, the flip angle correction is negligible; accordingly, it is ignored in the next sections.

## 3 Relaxation of hyperpolarized magnetization in ${}^{13}$ C substrates

We now take a detour into relaxation theory to give an overview of the factors that determine the values of $R_{1}=1/T_{1}$ of hyperpolarized ${}^{13}$ C solutes in a (bio)chemical system taking into account the main relaxation mechanisms responsible for the decay of the nuclear magnetization in the solution state at temperatures between ∼20 and 180 ${}^{\circ }$ C and static magnetic field strengths between 1 mT and 23.5 T. The spin interactions discussed here are relevant to the outcome of numerous dissolution-dynamic nuclear polarization (dDNP) experiments.

A master equation for spin systems far from equilibrium based on a Lindblad dissipator formalism has recently been presented and shown to correctly predict the spin dynamics of hyperpolarized systems (Bengs and Levitt, 2020). In brief, Eq. (2) is only valid for the high temperature limit and weak-order approximation of a spin system at thermal equilibrium. However, we do not pursue this line of enquiry here because for the enzyme systems studied thus far with dDNP a constant value of $T_{1}$ has been statistically satisfactory in regression analyses of the data (Pagès et al., 2013; Shishmarev et al., 2018b).

Once a sufficiently high level of nuclear spin polarization has been achieved by implementing dDNP methodologies (often for ${}^{13}$ C nuclei $P_{C}>60$ %), a jet of superheated solvent (e.g. H ${}_{2}$ O and/or D ${}_{2}$ O at 150–180 ${}^{\circ }$ C) is injected directly onto the hyperpolarized sample (Ardenkjaer-Larsen et al., 2003; Wolber et al., 2004). Upon contact with the warm solvent, the frozen sample rapidly dissolves and is then transferred under the pressure of helium gas (6–9 bar) to a separate NMR/MRI spectrometer for the detection of hyperpolarized MRS signals or to a collection/quality control point for use in biological applications (Comment and Merritt, 2014). There are several potential issues related to spin relaxation during these processes, and we focus on nuclear spin relaxation in solution during the sample transfer stage (i.e. subject to changes in magnetic field strength) or situations where a solute has an altered rotational correlation time (i.e. dependence on temperature or when bound to a protein). This requires an understanding of the (potentially) large variety of molecular interactions that give rise to nuclear spin relaxation.

- *Dipole–dipole couplings (DD).* The dominant mechanism for the relaxation of nuclear spin magnetization is often the stochastic modulation of dipole–dipole interactions (couplings) to other nuclei, either in the same molecule or other molecules, including the solvent, as the molecule re-orientates in solution by tumbling.
- *Chemical shift anisotropy (CSA).* Nuclear spins resonate at different frequencies depending on the chemical shielding imposed by the local electronic environment and its orientation (a tensor property). The modulation of the chemical shift tensor by molecular tumbling in solution has a quadratic dependence on the strength of the static magnetic field and therefore increases markedly with $B_{0}$ (Kowalewski and Maler, 2019).
- *Paramagnetic sites.* Dissolved paramagnetic solutes (often impurities, but they can be purposely added as required by the experimental design), such as radical agents that remain in the dissolution solvent, molecular oxygen, and metal ions, which can be deleterious to the nuclear-spin relaxation, particularly in regions of low magnetic field (Blumberg, 1960; Pell et al., 2019). However, all species can be easily scavenged by co-dissolving chelating agents in the dissolution medium (Mieville et al., 2010).
- *Scalar relaxation of the second kind.* This mechanism operates when the nuclei of interest have scalar couplings to neighbouring nuclei that also relax rapidly (Pileio, 2011; Kubica et al., 2014; Elliott et al., 2019). In dDNP NMR experiments this relaxation mechanism is often enhanced during sample transfer steps through areas of low magnetic field (Chiavazza et al., 2013; Kubica et al., 2014).
- *Spin rotation.* The coupling of nuclear magnetization to that of a whole molecule or to mobile parts of a molecule, e.g. methyl groups, can act as an efficient relaxation mechanism. This mechanism has an unusual dependence on temperature, with the relaxation rate usually increasing at higher temperatures (Matson, 1977).
- *Quadrupolar.* Many molecules of interest in dDNP experiments contain either ${}^{2}$ H or ${}^{14}$ N nuclei. NMR relaxation times of such nuclei are often <1 s and therefore not sufficiently long to be relevant for dDNP experiments. However, there are two notable exceptions in ${}^{6}$ Li ${}^{+}$ and ${}^{133}$ Cs ${}^{+}$ , which have small nuclear quadrupole moments and therefore have intrinsically long $T_{1}$ values (van Heeswijk et al., 2009; Kuchel et al., 2019).

Derivations of relaxation rate expressions are well established and based on plausible physical models. For simplicity, we skip the majority of these since they are comprehensively presented by several authors (Kowalewski and Maler, 2019), and instead we focus on the main results of their analyses. Assuming a two-spin system composed of a ${}^{13}$ C and ${}^{1}$ H, equations for the ${}^{13}$ C– ${}^{1}$ H dipole–dipole and the ${}^{13}$ C CSA contributions to the ${}^{13}$ C longitudinal relaxation rate constant ( $R_{1}$ ) are given by Keeler (Keeler, 2010):

26R1,DD=bHC2320JωC+120JωH-ωC+310JωH+ωC,27R1,CSA=c2115JωC,

where $b_{\mathrm{HC}}$ is the dipole–dipole coupling constant, defined as

28 $$b_{\mathrm{HC}}=\frac{\mu_{0}\gamma_{H}\gamma_{C}\hbar }{4\pi r_{\mathrm{HC}}^{3}},$$

and c is the magnitude of the CSA assuming an axially symmetrical tensor given by

29 $$c=\gamma_{C}B_{0}\left(\sigma_{∥}-\sigma_{⊥}\right),$$

where $\gamma_{H}$ and $\gamma_{C}$ are the magnetogyric ratios of the ${}^{1}$ H and ${}^{13}$ C spins, respectively, $r_{\mathrm{HC}}$ is the internuclear distance between the ${}^{1}$ H and ${}^{13}$ C atoms and $\sigma_{∥}$ and $\sigma_{⊥}$ are the parallel and perpendicular components of the axially symmetrical CSA tensor, respectively.

The so-called spectral density function that is a function of the Larmor frequency, ω , is

30 $$J\left(\omega \right)=\frac{2\tau_{c}}{1+\omega^{2}\tau_{c}^{2}},$$

where $\tau_{c}$ is the rotational correlation time (tumbling motion) of the re-orientating spin-bearing molecule in solution. The overall longitudinal relaxation rate constant is the sum of these two dominant contributions and is given by

31 $$R_{1}=R_{1,\mathrm{DD}}+R_{1,\mathrm{CSA}}.$$

### 3.1 Relaxation analysis

**Figure 3:**
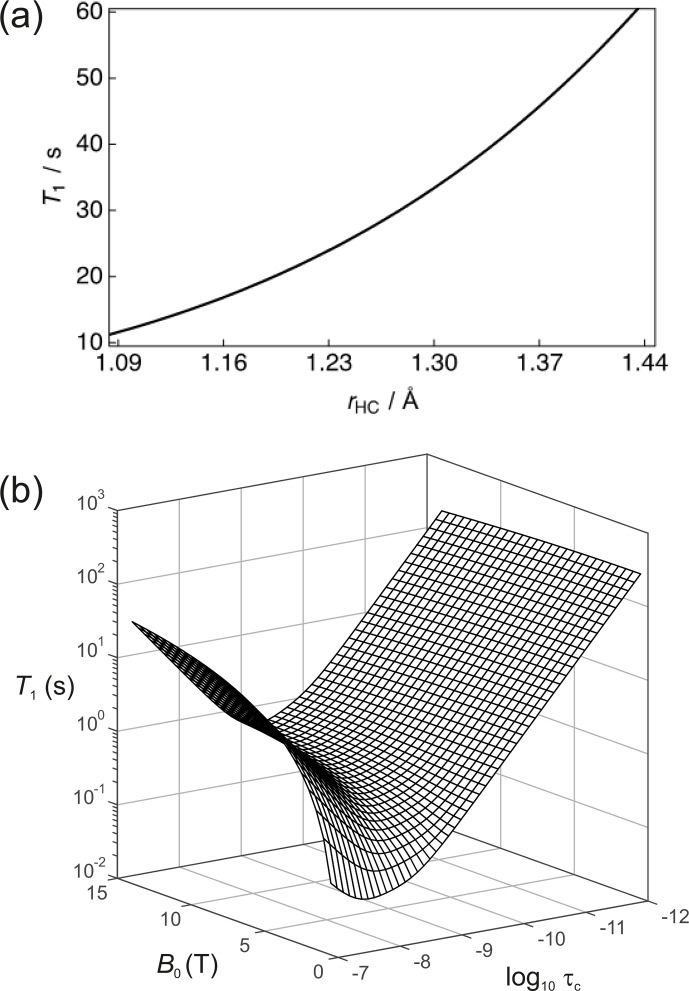
**(a)** Simulation of the ${}^{13}$ C nuclear $T_{1}$ for a two-spin ${}^{1}$ H– ${}^{13}$ C system as a function of the internuclear distance ( $r_{\mathrm{HC}}$ ) with a rotational correlation time $\tau_{c}=0.4\times 10^{-11}$ s, ${}^{13}$ C CSA $\sigma_{∥}-\sigma_{⊥}=-98$ ppm and at a magnetic field strength B=7 T. **(b)** Dependence of the ${}^{13}$ C nuclear $T_{1}$ as a function of the magnetic field B and the rotational correlation time $\tau_{c}$ .

It is important (for experimental design purposes) to note the influence that a nearby ${}^{1}$ H spin has on the ${}^{13}$ C nuclear $T_{1}$ . Figure 3a shows the calculated ${}^{13}$ C $T_{1}$ for a fixed rotational correlation time of $\tau_{c}=0.4\times 10^{-11}$ s (previously reported for glycine in saline at 310 K – Endre et al., 1983), ${}^{13}$ C CSA $\sigma_{∥}-\sigma_{⊥}=-98$ ppm (previously reported for phosphoenolpyruvate – Bechmann et al., 2004) and a magnetic field strength of $B_{0}=7$ T as a function of the ${}^{1}$ H– ${}^{13}$ C internuclear distance $r_{\mathrm{HC}}$ . Biaxiality of the CSA interaction has been ignored here. A rapid rise occurs in $T_{1}$ as the ${}^{1}$ H– ${}^{13}$ C internuclear separation increases. In the case of $r_{\mathrm{HC}}=1.09$ Å, which is typical of a ${}^{1}$ H– ${}^{13}$ C single bond, the ${}^{13}$ C nuclear $T_{1}$ is predicted to be ∼11.4 s. The ${}^{1}$ H– ${}^{13}$ C dipole–dipole coupling constant scales with $r_{\mathrm{HC}}^{-3}$ ; consequently, the presence of a directly bonded proton significantly shortens the relaxation time constant of the ${}^{13}$ C magnetization. Small molecules containing ${}^{13}$ C atoms that do not have directly bonded ${}^{1}$ H, or at least ${}^{1}$ H spins located at significant internuclear distances, are required. Such moieties include the carboxyl group that is present in many low molecular weight metabolites such as pyruvate, lactate, and methylglyoxal (Shishmarev et al., 2018a). At the longer ${}^{1}$ H– ${}^{13}$ C internuclear distance of 1.45 Å, implying a ${}^{1}$ H– ${}^{13}$ C dipole–dipole coupling constant of $b_{\mathrm{HC}}/2\pi =-10.2$ kHz, a ${}^{13}$ C nuclear $T_{1}$ of ∼60 s is predicted. At very long distances, the ${}^{13}$ C relaxation time constant will tend to that of the CSA relaxation contribution alone.

The dependence of $R_{1}$ on temperature and molecular size (e.g. due to binding) scales with the rotational correlation time. Figure 3b shows the dependence of the ${}^{13}$ C nuclear $T_{1}$ (1/ $R_{1}$ ) as a function of $\tau_{c}$ and $B_{0}$ for this 2-spin-1/2 system with $r_{\mathrm{HC}}=1.45$ Å and $\sigma_{∥}-\sigma_{⊥}=-98$ ppm. In the extreme narrowing limit, i.e. $\omega^{2}\tau_{c}^{2}\ll 1$ , the following familiar equations describe the relaxation of ${}^{13}$ C spins under the dipole–dipole and CSA relaxation mechanisms (Kowalewski and Maler, 2019):

32R1,DD=bHC2τc,33R1,CSA=215c2τc.

In the extreme narrowing regime the ${}^{13}$ C nuclear $T_{1}$ becomes shorter with increasing magnetic field strength due to the $B_{0}^{2}$ dependence of $R_{1,\mathrm{CSA}}$ . At low field strengths, the magnitude of $T_{1}$ will mostly be attributed to dipole–dipole relaxation with the nearby ${}^{1}$ H spin. It is also worth noting that the ${}^{13}$ C $T_{1}$ follows the usual Lorentzian spectral density functional dependence on the rotational correlation time. This is clearly seen at high magnetic fields.

### 3.2 Molecular considerations

The majority of dDNP experiments used to study biological systems employ $H_{2}O/D_{2}O$ as the dissolution solvent. Detection of hyperpolarized NMR/MRI signals typically occurs in a magnetic field range of 1.5–9.4 T; thus, Fig. 3b indicates a ${}^{13}$ C nuclear $T_{1}$ of the order of ∼60 s for a carbonyl group, and this is commonly seen in practice (Shishmarev et al., 2018a). It is important to remember that Eqs. (26)–(31) provide a greatly simplified picture of the problem at hand; in reality there are many magnetic nuclei (often within the same molecule) which contribute to the relaxation of ${}^{13}$ C magnetization. The additional dipole–dipole interactions are likely to be responsible for differences between predicted and measured ${}^{13}$ C relaxation times, along with the other (more exotic) signal attenuation mechanisms that are described above.

In a dDNP experiment the dissolution and transfer process can take as long as 15 s; it depends on the distance to the point of use from the polarizing source, and in clinical applications an additional 30 s can easily be added for quality control processes. Such requirements place a bound on the usable time in which hyperpolarized ${}^{13}$ C magnetization must be maintained, and it is typical to expect 45 s to be this limit. Given that the magnetic field strength “felt” by the hyperpolarized sample can be controlled (to a reasonable extent) throughout its voyage between the dDNP polarizer and the point of use (Milani et al., 2015), the rotational correlation time becomes the most important factor that impacts upon the ${}^{13}$ C nuclear $T_{1}$ . Figure 3b indicates that even for a rotational correlation time of the order of $\tau_{c}=1\times 10^{-10}$ s, such as found in proteins in solution (Wilbur et al., 1976), Eqs. (26)–(31) yield ${}^{13}$ C nuclear $T_{1}$ relaxation times which are too short to allow practical use of such samples, i.e. $5\times T_{1}\ll 45$ s, in comparison to the overall time required by a dDNP experiment.

A major parameter that controls the magnitude of the rotational correlation time of a spin-bearing molecule is its molecular weight ( $M_{w}$ ). Since $\tau_{c}\propto M_{w}$ the rotational correlation time has a noticeable impact on the ${}^{13}$ C nuclear $T_{1}$ with even the smallest increase in molecular weight. In order to achieve ${}^{13}$ C nuclear $T_{1}$ relaxation times that are sufficiently long to enable hyperpolarized ${}^{13}$ C magnetization to survive the dissolution and transfer process, the ${}^{13}$ C NMR signals must be detectable above the spectral noise for ∼45 s. Hence, dDNP samples used in biological experiments are currently restricted to small molecules (or ions – van Heeswijk et al., 2009; Kuchel et al., 2019). For example, the estimate of ∼60 s for the ${}^{13}$ C nuclear $T_{1}$ of the model system described above was predicted with a rotational correlation time of $\tau_{c}=0.4\times 10^{-11}$ (Endre et al., 1983), and this is sufficiently long for dDNP experiments.

### 3.3 Enzyme binding

The worst-case scenario for the model system described in Fig. 3b would be a moderate rotational correlation time of the order of $\tau_{c}=1\times 10^{-8}-1\times 10^{-10}$ s for which ${}^{13}$ C nuclear $T_{1}$ relaxation times in the millisecond regime are predicted. Such correlation times correspond to a system with a molecular weight comparable to that of an enzyme. If the small molecule (ligand) or ion becomes bound to the enzyme, then it will assume the rotational correlation time of the higher mass binding partner. In the case of $\tau_{c}=1\times 10^{-9}$ for an enzyme–ligand complex, a ${}^{13}$ C substrate will have a predicted nuclear magnetic $T_{1}$ of ∼276.4 ms at a static magnetic field strength of 7 T. Such a stark variation in ${}^{13}$ C nuclear $T_{1}$ values provides good contrast in relaxation-based ligand–protein binding experiments (Valensin et al., 1982).

## 4 Mechanistic description of reaction kinetics of hyperpolarized substrates

We now consider the interpretation of hyperpolarized dynamics for complex chemical reactions. To help tease apart the key features of the analysis, we begin with some simplifying assumptions. First, in the absence of an RF pulse Eq. (20) becomes block diagonal, since transverse and longitudinal magnetization are not interconverted. The evolution of the z magnetization is then dependent only on the initial conditions, $T_{1}$ , and the rate constants that characterize the chemical exchange. Second, we assume that the z magnetization is sampled many times with an infinitesimally small flip angle ( ≪1 ${}^{\circ }$ ) so the longitudinal magnetization decays with its intrinsic $T_{1}$ value rather than an apparent $T_{1,\mathrm{app}}$ value. Finally, the hyperpolarized magnetization decays to zero; i.e. the enhancement factor η (Eq. 21) is such that $M_{0}$ is greater than $M_{\mathrm{eq}}$ by many orders of magnitude. Thus, the equilibrium magnetization at t=∞ is effectively zero, and it can be ignored in the analysis of real experimental data.

To reduce clutter in the equations, for all the discussions that now follow, we drop the subscript z since we hereafter deal only with longitudinal magnetization and denote $M_{z,\mathrm{hyp}}^{A}$ and $M_{z,\mathrm{hyp}}^{B}$ as $A^{*}\left(t\right)$ and $B^{*}\left(t\right)$ corresponding to hyperpolarized magnetization (identified with an asterisk ${}^{*}$ ).

### 4.1 Simple first-order exchange kinetics of hyperpolarized substrates

Confining our analysis to the physical subspace that is composed of longitudinal magnetizations, which describe first-order kinetics of a two-site exchange reaction of hyperpolarized substrates, $A^{*}↔B^{*}$ , Eq. (20) simplifies to

34 $$\frac{d}{dt}\left[\begin{array}{c} A^{*}\left(t\right)\\ B^{*}\left(t\right) \end{array}\right]=\left[\begin{array}{cc} -k_{1}-R_{1}^{A} & k_{-1}\\ k_{1} & -k_{-1}-R_{1}^{B} \end{array}\right]\left[\begin{array}{c} A^{*}\left(t\right)\\ B^{*}\left(t\right) \end{array}\right].$$

Equivalently, Eq. (34) can be expanded to give

35dA∗tdt=-k1A∗t+k-1B∗t-R1AA∗t,36dB∗tdt=k1A∗t-k-1B∗t-R1BB∗t,

where $k_{1}$ and $k_{-1}$ denote first-order rate constants, and $R_{1}^{A}=1/T_{1}^{A}$ and $R_{1}^{B}=1/T_{1}^{B}$ are the longitudinal relaxation rate constants of A and B , respectively.

**Figure 4:**
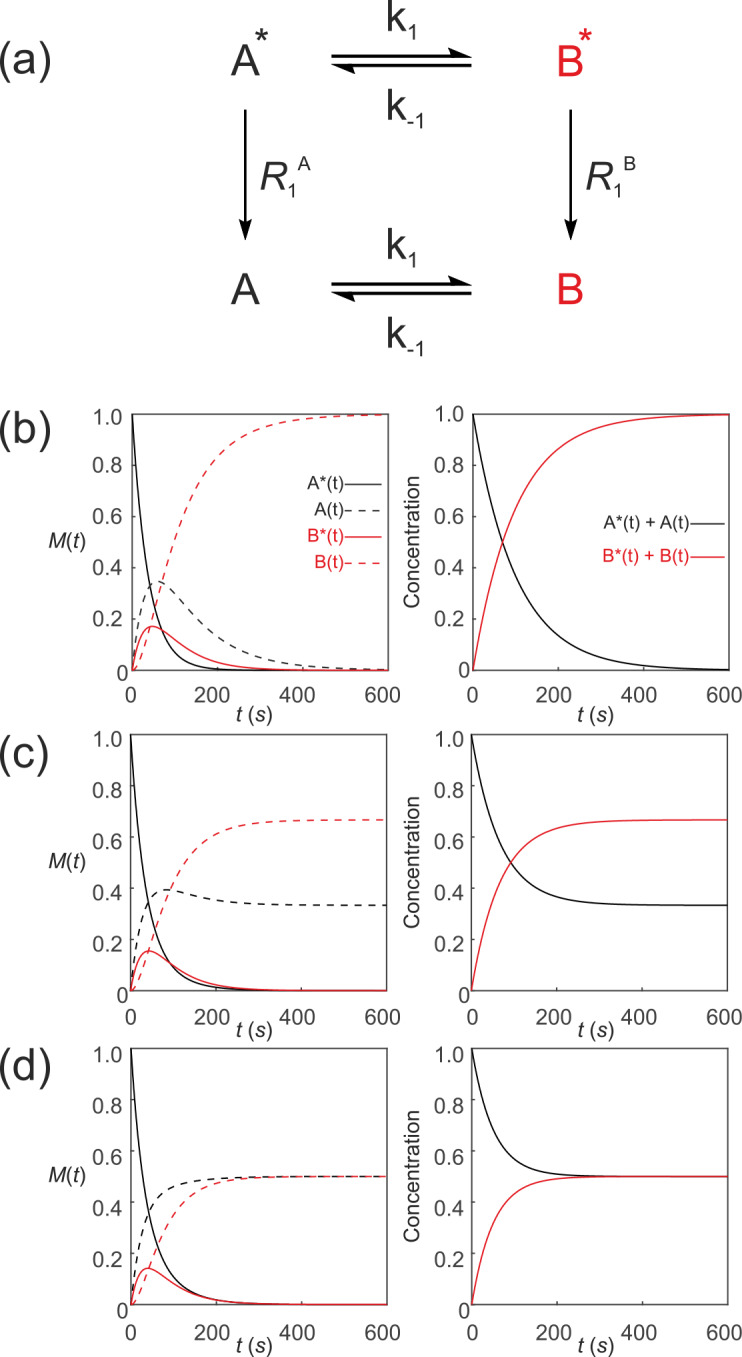
Simulated first-order two-site exchange kinetics of hyperpolarized solutes, $A^{*}↔B^{*}$ , conforming to conservation of mass, assuming initial hyperpolarized magnetization of only solute $A^{*}\left(0\right)=1$ . Longitudinal relaxation rate constants were $R_{1}^{A}=R_{1}^{B}=1/60$ s ${}^{-1}$ . The time dependences of $A^{*}\left(t\right)$ , $A\left(t\right)$ , $B^{*}\left(t\right)$ and $B\left(t\right)$ (left panel) were calculated numerically using Eq. (35)–(38) with rate constants **(b)** $k_{1}=0.01$ s ${}^{-1}$ , $k_{-1}=0$ s ${}^{-1}$ , corresponding to uni-directional kinetics, **(c)** $k_{1}=0.01$ s ${}^{-1}$ , $k_{-1}=0.005$ s ${}^{-1}$ and **(d)** $k_{1}=0.01$ s ${}^{-1}$ , $k_{-1}=0.01$ s ${}^{-1,}$ corresponding to exchange kinetics. The right panels show plots of the time dependence of $A^{*}\left(t\right)+A\left(t\right)=\left[A\left(t\right)\right]$ and $B^{*}\left(t\right)+B\left(t\right)=\left[B\left(t\right)\right]$ .

Since Eqs. (35) and (36) describe the time evolution of the z magnetizations (that is proportional to concentration/mass), they do not satisfy the conservation of mass requirement because $d\left[A^{*}\left(t\right)+B^{*}\left(t\right)\right]/dt=-R_{1}^{A}A^{*}\left(t\right)-R_{1}^{B}B^{*}\left(t\right)$ , and this tends to zero with time. However, the equations can be recast to specify that the pools of hyperpolarized substrates relax to form pools of non-polarized substrates A↔B . These pools are denoted simply by $A\left(t\right)$ and $B\left(t\right)$ (without the asterisks) as shown in Fig. 4a. The analogy with radioactive tracers is a useful one here. A “hot” pool of radioactive material decays with first-order kinetics (half-life) to form a “cold” pool of non-radioactive material with the sum of “hot” and “cold” being constant.

The kinetics of the non-polarized pools are described by

37dAtdt=-k1At+k-1Bt+R1AA∗t,38dBtdt=k1At-k-1Bt+R1BB∗t.

Equations (35)–(38) now satisfy conservation of mass, since the rate of change $d\left[A^{*}\left(t\right)+A\left(t\right)+B^{*}\left(t\right)+B\left(t\right)\right]/dt$ is always zero. Note that $A\left(t\right)$ and $B\left(t\right)$ are not observed in the dDNP NMR experiment, but they are the counterparts of real concentrations of solute that would be assayable (bio)chemically.

Equations (35)–(38) can be written as

39 $$\begin{array}{rl} & \frac{d}{dt}\left[\begin{array}{c} A^{*}\left(t\right)\\ B^{*}\left(t\right)\\ A\left(t\right)\\ B\left(t\right) \end{array}\right]=\\ & \left[\begin{array}{cccc} -k_{1}-R_{1}^{A} & k_{-1} & 0 & 0\\ k_{1} & -k_{-1}-R_{1}^{B} & 0 & 0\\ R_{1}^{A} & 0 & -k_{1} & k_{-1}\\ 0 & R_{1}^{B} & k_{1} & -k_{-1} \end{array}\right]\left[\begin{array}{c} A^{*}\left(t\right)\\ B^{*}\left(t\right)\\ A\left(t\right)\\ B\left(t\right) \end{array}\right]. \end{array}$$

Equation (39) can be written as

40 $$\frac{dM(t)}{dt}=LM(t).$$

We can apply a similarity transform given by

41 $$U=\left[\begin{array}{cccc} 1 & 0 & 0 & 0\\ 0 & 1 & 0 & 0\\ 1 & 0 & 1 & 0\\ 0 & 1 & 0 & 1 \end{array}\right],$$

to yield an equation of motion in a transformed vector basis

42 $$\frac{dM^{\prime }(t)}{dt}=ULU^{-1}M^{\prime }(t),$$

given by

43 $$\begin{array}{rl} & \frac{d}{dt}\left[\begin{array}{c} A^{*}\left(t\right)\\ B^{*}\left(t\right)\\ A^{*}\left(t\right)+A\left(t\right)\\ B^{*}\left(t\right)+B\left(t\right) \end{array}\right]=\\ & \left[\begin{array}{cccc} -k_{1}-R_{1}^{A} & k_{-1} & 0 & 0\\ k_{1} & -k_{-1}-R_{1}^{B} & 0 & 0\\ 0 & 0 & -k_{1} & k_{-1}\\ 0 & 0 & k_{1} & -k_{-1} \end{array}\right]\left[\begin{array}{c} A^{*}\left(t\right)\\ B^{*}\left(t\right)\\ A^{*}\left(t\right)+A\left(t\right)\\ B^{*}\left(t\right)+B\left(t\right) \end{array}\right]. \end{array}$$

We can now appreciate the equivalence between this formalism and conventional chemical reaction kinetics written in terms of molecular concentrations. For first-order reactions, the hyperpolarized magnetization evolves according to the Bloch–McConnell equations, while the concentrations given by the sum of the “hot” and “cold” pools evolve according to the conventional form of chemical reaction kinetics for a closed system. Therefore, $A^{*}\left(t\right)+A\left(t\right)$ and $B^{*}\left(t\right)+B\left(t\right)$ are proportional to [ A(t) ] and [ B(t) ], respectively, where the constant of proportionality depends on the initial experimental conditions viz. ${\left[A\right]}_{0}$ and ${\left[B\right]}_{0}$ . In other words, provided $A^{*}\left(0\right)+A\left(0\right)={\left[A\right]}_{0}$ and $B^{*}\left(0\right)+B\left(0\right)={\left[B\right]}_{0}$ , then the constant of proportionality is 1, and we can equate $A^{*}\left(t\right)+A\left(t\right)=\left[A\left(t\right)\right]$ and $B^{*}\left(t\right)+B\left(t\right)=\left[B\left(t\right)\right]$ . This is a crucial concept that we return to below.

Figure 4 shows numerical simulations of the time evolution of the system described by Eq. (39) with an initial magnetization vector $M\left(0\right)=\left[1,\;0,\;0,\;0\right]$ that corresponds to only hyperpolarized $A^{*}\left(0\right)=1$ and longitudinal relaxation rate constants $R_{1}^{A}=R_{1}^{B}=1/60$ s ${}^{-1}$ . The time dependence of $A^{*}\left(t\right)$ , $A\left(t\right)$ , $B^{*}\left(t\right)$ and $B\left(t\right)$ were calculated numerically (left panel) for different rate constants: Fig. 4b, $k_{1}=0.01$ s ${}^{-1}$ , $k_{-1}=0$ s ${}^{-1}$ , corresponding to a uni-directional reaction; Fig. 4c, $k_{1}=0.01$ s ${}^{-1}$ , $k_{-1}=0.005$ s ${}^{-1}$ , corresponding to bi-directional exchange with an equilibrium constant K=2 ; and Fig. 4d, $k_{1}=0.01$ s ${}^{-1}$ , $k_{-1}=0.01$ s ${}^{-1}$ , also corresponding to bi-directional exchange with an equilibrium constant K=1 . The right columns of plots show the time dependence of $A^{*}\left(t\right)+A\left(t\right)$ and $B^{*}\left(t\right)+B\left(t\right)$ that reproduce conventional kinetics of $\left[A\left(t\right)\right]$ and $\left[B\left(t\right)\right]$ , as required for mathematical and physical consistency.

The approach used here (as laid out in Kuchel and Shishmarev, 2020) enables us to create systems of differential equations that satisfy conservation of mass and therefore allow a study of the influence of non-hyperpolarized pools of substrates on reaction kinetics. The approach enables more complicated reaction mechanisms to be described to allow the inclusion of MR-invisible pools of substrates such as ${}^{12}$ C, which are known to affect the outcome of dDNP experiments in vivo. We consider some of these scenarios next.

### 4.2 Sequential reaction kinetics of hyperpolarized substrates

**Figure 5:**
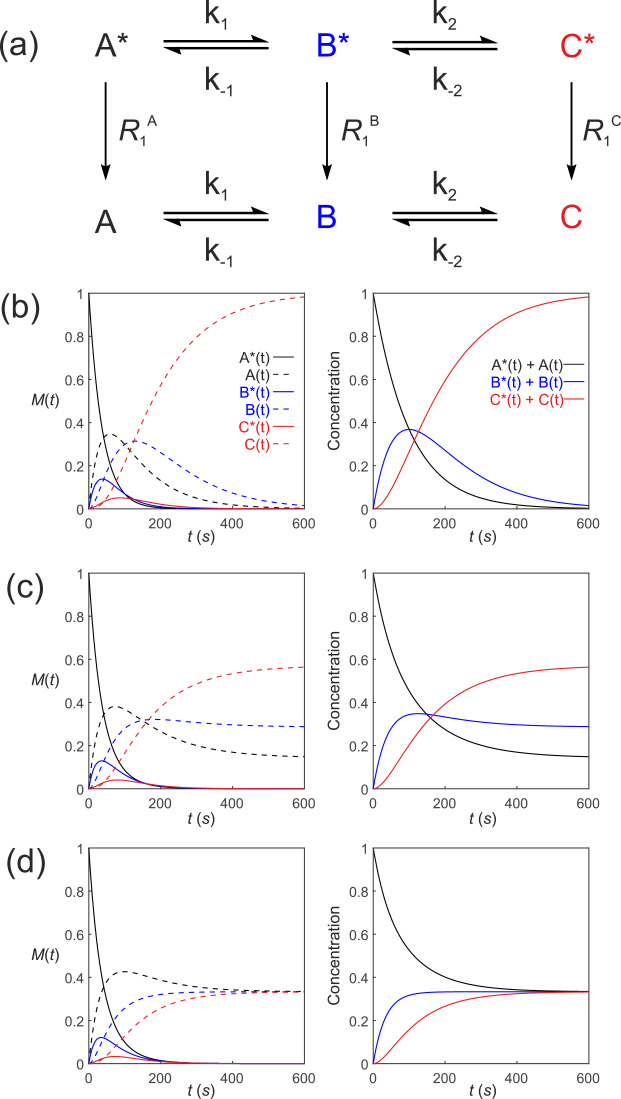
Simulated first-order three-site exchange kinetics of hyperpolarized solutes, $A^{*}↔B^{*}↔C^{*}$ , conforming to conservation of mass, assuming initial hyperpolarized magnetization of only solute $A^{*}\left(0\right)=1$ . Longitudinal relaxation rate constants were $R_{1}^{A}=R_{1}^{B}=R_{1}^{C}=1/60$ s ${}^{-1}$ . The time dependencies of $A^{*}\left(t\right)$ , $A\left(t\right)$ , $B^{*}\left(t\right)$ , $B\left(t\right)$ , $C^{*}\left(t\right)$ and $C\left(t\right)$ (left column of plots) were calculated numerically using Eqs. (41)–(46) with rate constants **(b)** $k_{1}=k_{2}=0.01$ s ${}^{-1}$ , $k_{-1}=k_{-2}=0$ s ${}^{-1}$ , corresponding to uni-directional kinetics, **(c)** $k_{1}=k_{2}=0.01$ s ${}^{-1}$ , $k_{-1}=k_{-2}=0.005$ s ${}^{-1}$ and **(d)** $k_{1}=k_{2}=k_{-1}=k_{-2}=0.01$ s ${}^{-1}$ , corresponding to exchange kinetics. The right column of plots shows the time dependence of $A^{*}\left(t\right)+A\left(t\right)=\left[A\left(t\right)\right]$ , $B^{*}\left(t\right)+B\left(t\right)=\left[B\left(t\right)\right]$ and $C^{*}\left(t\right)+C\left(t\right)=\left[C\left(t\right)\right]$ .

Equation (39) can be extended to compartmental models of arbitrary complexity: consider a reaction scheme involving three substrates $A^{*}↔B^{*}↔C^{*}$ which relax through $T_{1}$ processes to form a pool of non-polarized substrates A↔B↔C , as shown in Fig. 5a. This is analogous to a system where a solution of hyperpolarized solute $A^{*}$ is introduced into the extracellular medium in a cell suspension, is transported into the cells where it is denoted by $B^{*}$ and is subsequently acted upon by an enzyme to form $C^{*}$ . The system of differential equations that describe the kinetics of this scheme is

44dA∗tdt=-k1A∗t+k-1B∗t-R1AA∗t,45dB∗tdt=k1A∗t-k-1B∗t-k2B∗t+k-2C∗t-R1BB∗t,46dC∗tdt=k2B∗t-k-2C∗t-R1CC∗t,47dAtdt=-k1At+k-1Bt+R1AA∗t,48dBtdt=k1At-k-1Bt-k2Bt+k-2Ct+R1BB∗t,49dCtdt=k2Bt-k-2Ct+R1CC∗t,

where we have removed the square brackets that denote molar concentration to avoid some of the clutter. However, it is important to recall that there is a factor that relates magnetization to concentration, and this is estimated from the known initial experimental conditions.

Equations (44)–(49) can be recast in matrix form to give

50 $$\begin{array}{rl} & \frac{d}{dt}\left[\begin{array}{c} A^{*}\left(t\right)\\ B^{*}\left(t\right)\\ C^{*}\left(t\right)\\ A\left(t\right)\\ B\left(t\right)\\ C\left(t\right) \end{array}\right]=\\ & \left[\begin{array}{cccccc} -k_{1}-R_{1}^{A} & k_{-1} & 0 & 0 & 0 & 0\\ k_{1} & -k_{-1}-k_{2}-R_{1}^{B} & k_{-2} & 0 & 0 & 0\\ 0 & k_{2} & -k_{-2}-R_{1}^{C} & 0 & 0 & 0\\ R_{1}^{A} & 0 & 0 & -k_{1} & k_{-1} & 0\\ 0 & R_{1}^{B} & 0 & k_{1} & -k_{-1}-k_{2} & k_{-2}\\ 0 & 0 & R_{1}^{C} & 0 & k_{2} & -k_{-2} \end{array}\right]\\ & \left[\begin{array}{c} A^{*}\left(t\right)\\ B^{*}\left(t\right)\\ C^{*}\left(t\right)\\ A\left(t\right)\\ B\left(t\right)\\ C\left(t\right) \end{array}\right]. \end{array}$$

It is readily verified that Eq. (50) satisfies conservation of mass, since the rate of change $d\left(A^{*}\left(t\right)+A\left(t\right)+B^{*}\left(t\right)+B\left(t\right)+C^{*}\left(t\right)+C\left(t\right)\right)/dt=0$ .

We can apply a similarity transform given by

51 $$U=\left[\begin{array}{cccccc} 1 & 0 & 0 & 0 & 0 & 0\\ 0 & 1 & 0 & 0 & 0 & 0\\ 0 & 0 & 1 & 0 & 0 & 0\\ 1 & 0 & 0 & 1 & 0 & 0\\ 0 & 1 & 0 & 0 & 1 & 0\\ 0 & 0 & 1 & 0 & 0 & 1 \end{array}\right].$$

To yield an equation of motion in the transformed basis vector given by

52 $$\begin{array}{rl} & \frac{d}{dt}\left[\begin{array}{c} A^{*}\left(t\right)\\ B^{*}\left(t\right)\\ C^{*}\left(t\right)\\ A^{*}\left(t\right)+A\left(t\right)\\ B^{*}\left(t\right)+B\left(t\right)\\ C^{*}\left(t\right)+C\left(t\right) \end{array}\right]=\\ & \left[\begin{array}{cccccc} -k_{1}-R_{1}^{A} & k_{-1} & 0 & 0 & 0 & 0\\ k_{1} & -k_{-1}-k_{2}-R_{1}^{B} & k_{-2} & 0 & 0 & 0\\ 0 & k_{2} & -k_{-2}-R_{1}^{C} & 0 & 0 & 0\\ 0 & 0 & 0 & -k_{1} & k_{-1} & 0\\ 0 & 0 & 0 & k_{1} & -k_{-1}-k_{2} & k_{-2}\\ 0 & 0 & 0 & 0 & k_{2} & -k_{-2} \end{array}\right]\\ & \left[\begin{array}{c} A^{*}\left(t\right)\\ B^{*}\left(t\right)\\ C^{*}\left(t\right)\\ A^{*}\left(t\right)+A\left(t\right)\\ B^{*}\left(t\right)+B\left(t\right)\\ C^{*}\left(t\right)+C\left(t\right) \end{array}\right], \end{array}$$

the hyperpolarized magnetization evolves according to the Bloch–McConnell equations, while the concentrations given by the sum of the “hot” and “cold” pools evolve according to the conventional form of chemical reaction kinetics for a closed system. Therefore, provided $A^{*}\left(0\right)+A\left(0\right)={\left[A\right]}_{0}$ , $B^{*}\left(0\right)+B\left(0\right)={\left[B\right]}_{0}$ and $C^{*}\left(0\right)+C\left(0\right)={\left[C\right]}_{0}$ , then $A^{*}\left(t\right)+A\left(t\right)=\left[A\left(t\right)\right]$ , $B^{*}\left(t\right)+B\left(t\right)=\left[B\left(t\right)\right]$ and $C^{*}\left(t\right)+C\left(t\right)=\left[C\left(t\right)\right]$ , respectively.

Figure 5 shows the results of numerical integration of Eq. (50) with the initial magnetization vector $M\left(0\right)=\left[1,0,0,0,0,0\right]$ that corresponds to having only hyperpolarized $A^{*}(0)=1$ and longitudinal relaxation rate constants $R_{1}^{A}=R_{1}^{B}=R_{1}^{C}=1/60$ s ${}^{-1}$ . The time dependence of $A^{*}\left(t\right)$ , $A\left(t\right)$ , $B^{*}\left(t\right)$ , $B\left(t\right)$ , $C^{*}\left(t\right)$ and $C\left(t\right)$ were calculated (left panel) for different rate constants: Fig. 5b, $k_{1}=k_{2}=0.01$ s ${}^{-1}$ , $k_{-1}=k_{-2}=0$ s ${}^{-1}$ , corresponding to uni-directional kinetics; Fig. 5c, $k_{1}=k_{2}=0.01$ s ${}^{-1}$ , $k_{-1}=k_{-2}=0.005$ s ${}^{-1}$ , corresponding to bi-directional exchange kinetics; and Fig. 5d, $k_{1}=k_{2}=k_{-1}=k_{-2}=0.01$ s ${}^{-1}$ , also corresponding to bi-directional exchange kinetics. The right column shows plots of the time dependence of $A^{*}\left(t\right)+A\left(t\right)$ , $B^{*}\left(t\right)+B\left(t\right)$ and $C^{*}\left(t\right)+C\left(t\right)$ , which reproduce the conventional chemical kinetics of $\left[A\left(t\right)\right]$ , $\left[B\left(t\right)\right]$ and $\left[C\left(t\right)\right]$ , as required for mathematical and physical consistency.

### 4.3 Second-order kinetics of hyperpolarized substrates

**Figure 6:**
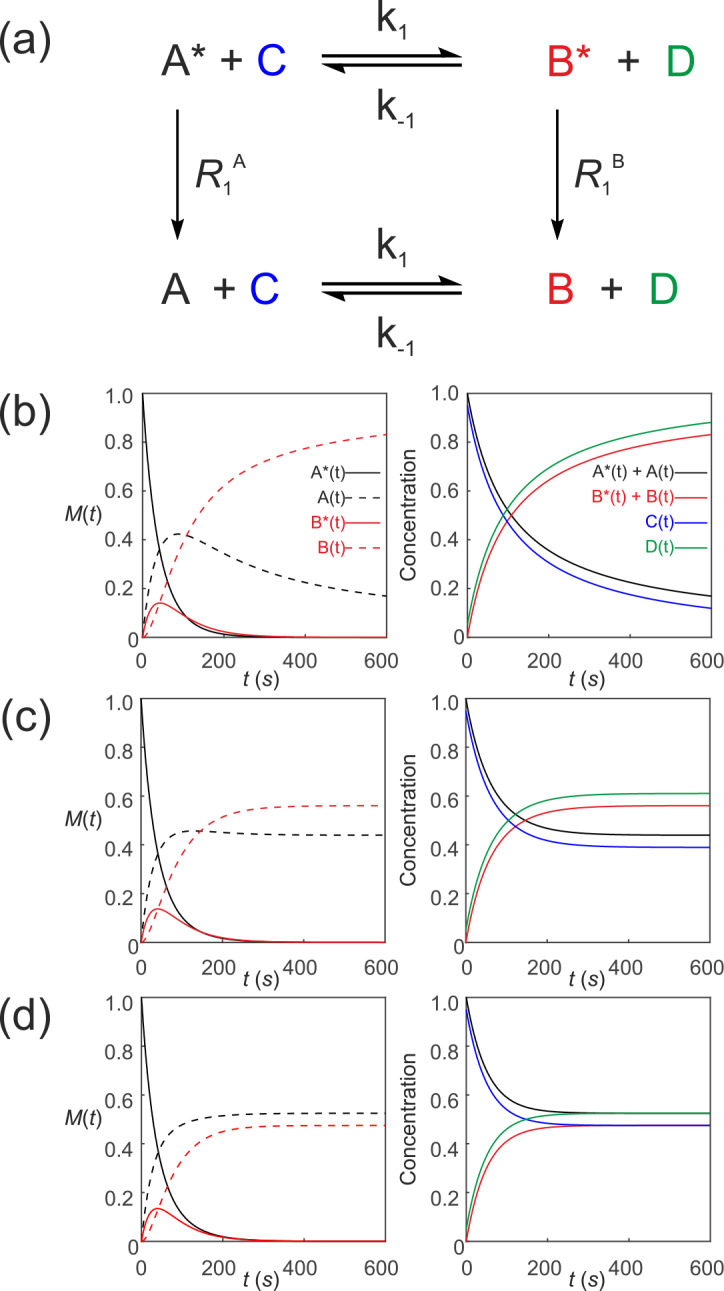
Simulated second-order exchange kinetics of hyperpolarized solutes, $A^{*}+C↔B^{*}+D$ , conforming to conservation of mass, assuming initial hyperpolarized magnetization of only solute $A^{*}\left(0\right)=1$ . Longitudinal relaxation rate constants were $R_{1}^{A}=R_{1}^{B}=1/60$ s ${}^{-1}$ . The time dependences of $A^{*}\left(t\right)$ , $A\left(t\right)$ , $B^{*}\left(t\right)$ and $B\left(t\right)$ were simulated (left column of plots) using Eqs. (53)–(58) with rate constants **(b)** $k_{1}=0.01$ M ${}^{-1}$ s ${}^{-1}$ , $k_{-1}=0$ M ${}^{-1}$ s ${}^{-1}$ , corresponding to uni-directional kinetics **(c)** $k_{1}=0.01$ M ${}^{-1}$ s ${}^{-1}$ , $k_{-1}=0.005$ M ${}^{-1}$ s ${}^{-1}$ and **(d)** $k_{1}=k_{-1}=0.01$ M ${}^{-1}$ s ${}^{-1}$ , corresponding to exchange kinetics. The right column of plots shows the time dependence of $A^{*}\left(t\right)+A\left(t\right)=\left[A\left(t\right)\right]$ , $B^{*}\left(t\right)+B\left(t\right)=\left[B\left(t\right)\right]$ and non-polarized reactants $\left[C\left(t\right)\right]$ and $\left[D\left(t\right)\right]$ .

We now describe hyperpolarized substrates $A^{*}\left(t\right)$ and $B^{*}\left(t\right)$ reacting with non-hyperpolarized substrates $\left[C\left(t\right)\right]$ and $\left[D\left(t\right)\right]$ . The system of differential equations describes the second-order kinetics of $A^{*}+C↔B^{*}+D$ with only the hyperpolarized pools relaxing through $T_{1}$ processes to form a pool of non-polarized substrates A+C↔B+D . The reactant concentrations $\left[C\left(t\right)\right]$ and $\left[D\left(t\right)\right]$ are common to both pools, as shown in Fig. 6a. The relevant system of differential equations (again omitting the square brackets that denote concentration) is

53dA∗tdt=-k1CtA∗t+k-1DtB∗t-R1AA∗t,54dB∗tdt=k1CtA∗t-k-1DtB∗t-R1BB∗t,55dAtdt=-k1CtAt+k-1DtBt+R1AA∗t,56dBtdt=k1CtAt-k-1DtBt+R1BB∗t,57dCtdt=-k1A∗t+AtCt+k-1B∗t+BtDt,58dDtdt=k1A∗t+AtCt-k-1B∗t+BtDt.

Again, mass is conserved, as seen by the fact that $d((A^{*}\left(t\right)+A\left(t\right)+B^{*}\left(t\right)+B\left(t\right))/dt=0$ and $d\left(C\left(t\right)+D\left(t\right)\right)/dt=0$ . Also, recall that, provided $A^{*}\left(0\right)+A\left(0\right)={\left[A\right]}_{0}$ , $B^{*}\left(0\right)+B\left(0\right)={\left[B\right]}_{0}$ , $C\left(0\right)={\left[C\right]}_{0}$ and $D\left(0\right)={\left[D\right]}_{0}$ , then we can make use of the equalities $A^{*}\left(t\right)+A\left(t\right)=\left[A\left(t\right)\right]$ , $B^{*}\left(t\right)+B\left(t\right)=\left[B\left(t\right)\right]$ , $C\left(t\right)=\left[C\left(t\right)\right]$ and $D\left(t\right)=\left[D\left(t\right)\right]$ , respectively. It is now very evident why we must equate the initial signal with the concentration via an experimentally estimated scaling factor.

Equations (53)–(58) can be written in matrix vector form as

59 $$\begin{array}{rl} & \frac{d}{dt}\left[\begin{array}{c} A^{*}\left(t\right)\\ B^{*}\left(t\right)\\ A\left(t\right)\\ B\left(t\right)\\ C\left(t\right)\\ D\left(t\right) \end{array}\right]=\\ & \left[\begin{array}{cccccc} -k_{1}C\left(t\right)-R_{1}^{A} & k_{-1}D\left(t\right) & 0 & 0 & 0 & 0\\ k_{1}C\left(t\right) & -k_{-1}D\left(t\right)-R_{1}^{B} & 0 & 0 & 0 & 0\\ R_{1}^{A} & 0 & -k_{1}C\left(t\right) & k_{-1}D\left(t\right) & 0 & 0\\ 0 & R_{1}^{B} & k_{1}C\left(t\right) & -k_{-1}D\left(t\right) & 0 & 0\\ -k_{1}C\left(t\right) & k_{-1}D\left(t\right) & -k_{1}C\left(t\right) & k_{-1}D\left(t\right) & 0 & 0\\ k_{1}C\left(t\right) & -k_{-1}D\left(t\right) & k_{1}C\left(t\right) & -k_{-1}D\left(t\right) & 0 & 0 \end{array}\right]\\ & \left[\begin{array}{c} A^{*}\left(t\right)\\ B^{*}\left(t\right)\\ A\left(t\right)\\ B\left(t\right)\\ C\left(t\right)\\ D\left(t\right) \end{array}\right]. \end{array}$$

We can apply a similarity transform given by

60 $$U=\left[\begin{array}{cccccc} 1 & 0 & 0 & 0 & 0 & 0\\ 0 & 1 & 0 & 0 & 0 & 0\\ 1 & 0 & 1 & 0 & 0 & 0\\ 0 & 1 & 0 & 1 & 0 & 0\\ 0 & 0 & 0 & 0 & 1 & 0\\ 0 & 0 & 0 & 0 & 0 & 1 \end{array}\right],$$

to yield an equation of motion in the transformed basis vector given by

61 $$\begin{array}{rl} & \frac{d}{dt}\left[\begin{array}{c} A^{*}\left(t\right)\\ B^{*}\left(t\right)\\ A^{*}\left(t\right)+A\left(t\right)\\ B^{*}\left(t\right)+B\left(t\right)\\ C\left(t\right)\\ D\left(t\right) \end{array}\right]=\\ & \left[\begin{array}{cccccc} -k_{1}C\left(t\right)-R_{1}^{A} & k_{-1}D\left(t\right) & 0 & 0 & 0 & 0\\ k_{1}C\left(t\right) & k_{-1}D\left(t\right)-R_{1}^{B} & 0 & 0 & 0 & 0\\ 0 & 0 & -k_{1}C\left(t\right) & k_{-1}D\left(t\right) & 0 & 0\\ 0 & 0 & k_{1}C\left(t\right) & -k_{-1}D\left(t\right) & 0 & 0\\ 0 & 0 & -k_{1}C\left(t\right) & k_{-1}D\left(t\right) & 0 & 0\\ 0 & 0 & k_{1}C\left(t\right) & -k_{-1}D\left(t\right) & 0 & 0 \end{array}\right]\\ & \left[\begin{array}{c} A^{*}\left(t\right)\\ B^{*}\left(t\right)\\ A^{*}\left(t\right)+A\left(t\right)\\ B^{*}\left(t\right)+B\left(t\right)\\ C\left(t\right)\\ D\left(t\right) \end{array}\right]. \end{array}$$

Figure 6 shows numerical simulations of the time evolution of the system of Eqs. (53)–(58) with initial magnetization corresponding to the hyperpolarized signal $A^{*}(0)=1$ and non-polarized substrates $C\left(0\right)=0.95$ and $D\left(0\right)=0.05$ . The longitudinal relaxation rate constants were $R_{1}^{A}=R_{1}^{B}=1/60$ s ${}^{-1}$ . The time dependences of $A^{*}\left(t\right)$ , $A\left(t\right)$ , $B^{*}\left(t\right)$ and $B\left(t\right)$ are subject to second-order kinetics and were calculated numerically (left column of plots) for different rate constants: Fig. 6b, $k_{1}=0.01$ M ${}^{-1}$ s ${}^{-1}$ , $k_{-1}=0$ M ${}^{-1}$ s ${}^{-1}$ , corresponding to uni-directional kinetics; Fig. 6c, $k_{1}=0.01$ M ${}^{-1}$ s ${}^{-1}$ , $k_{-1}=0.005$ M ${}^{-1}$ s ${}^{-1}$ , corresponding to bi-directional exchange kinetics with an equilibrium constant K=2 ; and Fig. 6d $k_{1}=k_{-1}=0.01$ M ${}^{-1}$ s ${}^{-1}$ , with an equilibrium constant K=1 , also corresponding to bi-directional exchange kinetics. The right column of plots shows the time dependence of $A^{*}\left(t\right)+A\left(t\right)$ , $B^{*}\left(t\right)+B\left(t\right)$ , which capture conventional chemical kinetics of the concentrations of $\left[A\left(t\right)\right]$ and $\left[B\left(t\right)\right]$ , as expected, as well as the kinetics of the non-polarized reactants $\left[C\left(t\right)\right]$ and $\left[D\left(t\right)\right]$ .

#### 4.3.1 An ersatz solution

The system of differential equations in Eq. (59) describing a second-order reaction can be reduced to one with pseudo first-order kinetics by introducing time-dependent rate constants $k_{1}^{\prime }\left(t\right)=k_{1}C\left(t\right)$ and $k_{-1}^{\prime }\left(t\right)=k_{-1}\;D\left(t\right)$ . Importantly, the pseudo first-order rate constants $k_{1}^{\prime }\left(t\right)$ and $k_{-1}^{\prime }\left(t\right)$ are now time-dependent. This approach has been used previously (Mariotti et al., 2016), but it constitutes a special case of the more general method described here, which we now advocate.

However, we now encounter a problem. The pseudo first-order rate constants for the reactions of [ C(t) ] and [ D(t) ] are now given by $k_{1}^{\prime }\left(t\right)=k_{1}\left(A^{*}\left(t\right)+A\left(t\right)\right)$ and $k_{-1}^{\prime }\left(t\right)=k_{-1}\left(B^{*}\left(t\right)+B\left(t\right)\right)$ , respectively. The time-dependent pseudo first-order rate constants are dependent on the concentrations of both “hot” *and* “cold” pools. In turn, the pseudo first-order rate constants for $A^{*}\left(t\right)$ and $B^{*}\left(t\right)$ are $k_{1}^{\prime }\left(t\right)=k_{1}C(t)$ and $k_{-1}^{\prime }\left(t\right)=k_{-1}D(t)$ . Thus, the kinetics of the “hot” pools $A^{*}\left(t\right)$ and $B^{*}\left(t\right)$ become dependent on the kinetics of the “cold” pools $A\left(t\right)$ and $B\left(t\right)$ . This is of particular relevance (as highlighted by Kuchel and Shishmarev, 2020) when extending the equations to describe enzyme kinetics. It is this that we turn our attention to next.

## 5 Michaelis–Menten equation for a hyperpolarized substrate

**Figure 7:**
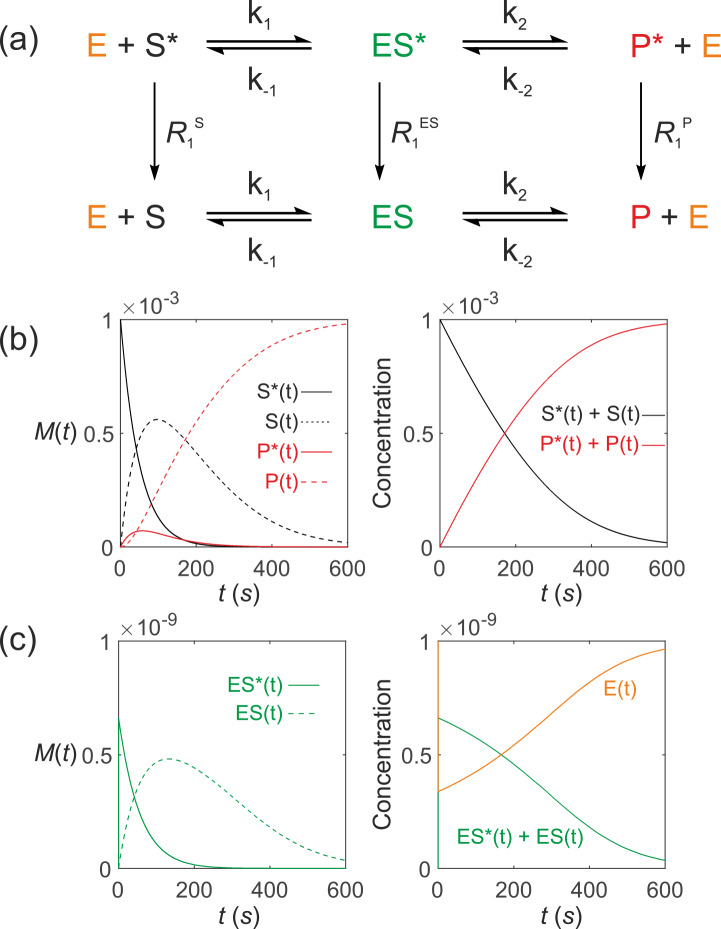
Simulated Michaelis–Menten kinetics for exchange of hyperpolarized solutes $E+S^{*}↔{\mathrm{ES}}^{*}↔P^{*}+E$ conforming to conservation of mass, assuming initial hyperpolarized magnetization of only solute $S^{*}\left(0\right)=0.001$ and ${\left[E\right]}_{0}=1\times 10^{-9}$ M. Longitudinal relaxation rate constants were $R_{1}^{S}=R_{1}^{\mathrm{ES}}=R_{1}^{P}=1/60$ s ${}^{-1}$ . The reaction rate constants were $k_{1}=1\times 10^{7}$ M ${}^{-1}$ s ${}^{-1}$ , $k_{-1}=1\times 10^{2}$ s ${}^{-1}$ , $k_{2}=5\times 10^{3}$ s ${}^{-1}$ and $k_{-2}=0$ M ${}^{-1}$ s ${}^{-1}$ , such that $K_{M}=5.1\times 10^{-4}$ M and $V_{\max}=5\times 10^{-6}$ M s ${}^{-1}$ . Left panels: **(b)** simulated time dependence of $S^{*}\left(t\right)$ , $S\left(t\right)$ , $P^{*}\left(t\right)$ and $P\left(t\right)$ , and **(c)** simulated time dependence of ${\mathrm{ES}}^{*}\left(t\right)$ and $\mathrm{ES}\left(t\right)$ . Right panels:** (b)** simulated time dependence of $S^{*}\left(t\right)+S\left(t\right)=\left[S\left(t\right)\right]$ and $P^{*}\left(t\right)+P\left(t\right)=\left[P\left(t\right)\right]$ , and **(c)** ${\mathrm{ES}}^{*}\left(t\right)+\mathrm{ES}\left(t\right)=\left[\mathrm{ES}\left(t\right)\right]$ and $\left[E\left(t\right)\right]$ .

Next consider an enzyme-catalysed reaction with a hyperpolarized substrate. The simplest model involves a hyperpolarized substrate $S^{*}\left(t\right)$ that is in equilibrium with a free enzyme of concentration ${\left[E\right]}_{0}$ to form a hyperpolarized enzyme–substrate complex ${\mathrm{ES}}^{*}\left(t\right)$ , which then reacts to form a hyperpolarized product $P^{*}(t)$ . This is followed by release of the free enzyme that is then available for further reactions: $E+S^{*}↔{\mathrm{ES}}^{*}↔P^{*}+E$ . All hyperpolarized substrates relax through $T_{1}$ processes to form non-polarized pools of substrates E+S↔ES↔P+E as shown in Fig. 7a. The differential equations (again omitting the square brackets denoting concentration) that describe the reaction kinetics are

62dS∗tdt=-k1EtS∗t+k-1ES∗t-R1SS∗t,63dES∗tdt=k1EtS∗t-k-1ES∗t-k2ES∗t+k-2EtP∗t-R1ESES∗t,64dP∗tdt=k2ES∗t-k-2EtP∗t-R1PP∗t,65dStdt=-k1EtSt+k-1ESt+R1SS∗t,66dEStdt=k1EtSt-k-1ESt-k2ESt+k-2EtPt+R1ESES∗t,67dPtdt=k2ESt-k-2EtPt+R1PP∗t,68dEtdt=-k1EtS∗t+St+k-1+k2ES∗t+ESt-k-2EtP∗t+Pt,

where $E\left(t\right)$ is the free enzyme, ES $\left(t\right)$ is the enzyme–substrate complex, $S\left(t\right)$ is the free substrate and $P\left(t\right)$ is the free product, with relaxation rate constants $R_{1}^{S}$ , $R_{1}^{\mathrm{ES}}$ and $R_{1}^{P}$ , respectively. Note the appearance of the free enzyme E(t) as both a reactant and product; it is regenerated through the reactions that are characterized by the rate constants $k_{1}$ and $k_{-1}$ and also $k_{2}$ and $k_{-2}$ , thereby being recycled.

Mass is conserved as confirmed by the fact that $d\left(S^{*}\left(t\right)+S\left(t\right)+{\mathrm{ES}}^{*}\left(t\right)+\mathrm{ES}\left(t\right)+P^{*}\left(t\right)+P\left(t\right)\right)/dt=0$ and $d\left({\mathrm{ES}}^{*}\left(t\right)+\mathrm{ES}\left(t\right)+E\left(t\right)\right)/dt=0$ . Therefore, provided $S^{*}\left(0\right)+S\left(0\right)={\left[S\right]}_{0}$ , ${\mathrm{ES}}^{*}\left(0\right)+\mathrm{ES}\left(0\right)={\left[\mathrm{ES}\right]}_{0}$ and $P^{*}\left(0\right)+P\left(0\right)={\left[P\right]}_{0}$ , then $S^{*}\left(t\right)+S\left(t\right)=\left[S\left(t\right)\right]$ , ${\mathrm{ES}}^{*}\left(t\right)+\mathrm{ES}\left(t\right)=\left[\mathrm{ES}\left(t\right)\right]$ and $P^{*}\left(t\right)+P\left(t\right)=\left[P\left(t\right)\right]$ , respectively.

Equations (62)–(68) can be written in matrix vector form given by Eq. (A69); see Appendix. We can apply a similarity transform, given by Eq. (A70) (see Appendix), to yield an equation of motion in the transformed basis vector given by Eq. (A71); see Appendix.

### 5.1 Steady state of ES complex

A simplified uni-directional enzyme-catalysed reaction is described by setting the reverse rate constant $k_{-2}=0$ (see Fig. 7a). If it is assumed that a steady state of [ES] is attained very rapidly, then $d\left({\mathrm{ES}}^{*}\left(t\right)+\mathrm{ES}\left(t\right)\right)/dt=0$ , and we obtain (reverting to using square brackets to denote molar concentration)

72 $$k_{1}\left[E\left(t\right)\right]\left[S^{*}\left(t\right)+S\left(t\right)\right]=\left(k_{-1}+k_{2}\right)\left[{\mathrm{ES}}^{*}\left(t\right)+\mathrm{ES}\left(t\right)\right].$$

Rearranging Eq. (72) yields the Michaelis constant in terms of hyperpolarized and non-polarized pools of substrate:

73 $$K_{M}=\frac{\left(k_{-1}+k_{2}\right)}{k_{1}}=\frac{\left[E\left(t\right)\right]\left[S^{*}\left(t\right)+S\left(t\right)\right]}{\left[{\mathrm{ES}}^{*}\left(t\right)+\mathrm{ES}\left(t\right)\right]}.$$

Calibrating the signals to molar concentrations is important since the signals now relate to a real parameter ( $K_{M}$ ) of the enzyme that has units of concentration (typically mM).

Thus, using conservation of enzyme mass, the free enzyme concentration is given by

74 $$[E(t)]=[E{]}_{0}-\left[{\mathrm{ES}}^{*}\left(t\right)+\mathrm{ES}\left(t\right)\right].$$

Then

75 $$\frac{d\left(\left[P^{*}\left(t\right)+P\left(t\right)\right]\right)}{dt}=\frac{k_{2}[E{]}_{0}\;[S^{*}\left(t\right)+S(t)]}{K_{M}+\left[S^{*}\left(t\right)+S\left(t\right)\right]}.$$

In other words, this is the standard form of the Michaelis–Menten equation written as a function of both polarized and unpolarized pools of substrate.

### 5.2 Simulations of the Michaelis–Menten reaction

Figure 7b–c show the results of numerical integration of Eqs. (62)–(68) with an initial hyperpolarized signal $S^{*}\left(0\right)=0.001$ (corresponding to a concentration ${\left[S\right]}_{0}=1$ mM via the experimentally determined scaling factor, which here was set to 1) and enzyme concentration ${\left[E\right]}_{0}=1\times 10^{-9}$ M. The assigned longitudinal relaxation rate constants were $R_{1}^{S}=R_{1}^{\mathrm{ES}}=R_{1}^{P}=1/60$ s ${}^{-1}$ . In the first instance, we set the longitudinal relaxation times of substrate, enzyme–substrate complex and product to be equal (this is discussed further below). The reaction rate constants were $k_{1}=1\times 10^{7}$ M ${}^{-1}$ s ${}^{-1}$ , $k_{-1}=1\times 10^{2}$ s ${}^{-1}$ , $k_{2}=5\times 10^{3}$ s ${}^{-1}$ , and $k_{-2}=0$ M ${}^{-1}$ s ${}^{-1}$ , such that $K_{M}=5.1\times 10^{-4}$ M and $V_{\max}=5\times 10^{-6}$ M s ${}^{-1}$ . The time dependences of $S^{*}\left(t\right)$ , $S\left(t\right)$ , $P^{*}\left(t\right)$ and $P\left(t\right)$ are shown in Fig. 7b, left panel, subject to standard uni-directional Michaelis–Menten kinetics, and in Fig. 7c, left panel, the time dependence of ${\mathrm{ES}}^{*}\left(t\right)$ and ES $\left(t\right)$ . The time dependence of $S^{*}\left(t\right)+S\left(t\right)=\left[S\left(t\right)\right]$ and $P^{*}\left(t\right)+P\left(t\right)=\left[P\left(t\right)\right]$ are shown in Fig. 7b, right panel, and ${\mathrm{ES}}^{*}\left(t\right)+\mathrm{ES}\left(t\right)=\left[\mathrm{ES}\left(t\right)\right]$ and $\left[E\left(t\right)\right]$ are shown in Fig. 7c, right panel, which recapture conventional chemical kinetics of $\left[S\left(t\right)\right]$ , $\left[\mathrm{ES}\left(t\right)\right]$ , $\left[P\left(t\right)\right]$ and $\left[E\left(t\right)\right]$ , as required for mathematical and physical consistency.

It is worth considering some of the consequences of Eq. (75) when studying enzyme-mediated reactions with hyperpolarized substrates. When the substrate concentration $\left[S^{*}\left(t\right)+S\left(t\right)\right]$ is much greater than $K_{M}$ , then the rate of product formation $d\left(\left[P^{*}\left(t\right)+P\left(t\right)\right]\right)/dt$ is given by $v=k_{2}{\left[E\right]}_{0}=V_{\max}$ , which is constant (i.e. it is effectively a zero-order reaction with respect to substrate concentration). The enzyme is said to be saturated; its rate is independent of substrate concentration, but $V_{\max}$ is proportional to the enzyme concentration ${\left[E\right]}_{0}$ . When the substrate concentration $\left[S^{*}\left(t\right)+S\left(t\right)\right]$ is much less than $K_{M}$ , then the rate of product formation $d\left(\left[P^{*}\left(t\right)+P\left(t\right)\right]\right)/dt$ is given by $V=k_{2}{\left[E\right]}_{0}\left[S^{*}\left(t\right)+S\left(t\right)\right]/K_{M}$ and the reaction is effectively first order with respect to substrate concentration. Nevertheless, the rate is still proportional to ${\left[E\right]}_{0}$ as well. The kinetics of enzyme systems, and indeed enzyme kinetics in general, are a composite of the two parameters $K_{M}$ and $V_{\max}$ . The influences on one cannot be distinguished from the other on the basis of time course experiments alone; separate measurements are needed to estimate the total enzyme concentration.

Further simulations were performed to explore the influence of a much shorter value of $T_{1}^{\mathrm{ES}}$ for the enzyme–substrate complex, while $T_{1}^{S}$ and $T_{1}^{P}$ were unchanged. Even if it were assumed to be very small viz. $T_{1}^{\mathrm{ES}}=276.4$ ms, the time evolution was indistinguishable from that presented in Fig. 7; the corresponding curves were superimposable. The signal that resided on the enzyme–substrate complex ${\mathrm{ES}}^{*}$ was 6 orders of magnitude lower than that of the substrate $S^{*}$ and product $P^{*}$ . Therefore, the kinetics of signal evolution were dominated by $T_{1}^{S}$ and $T_{1}^{P}$ , while changes in $T_{1}^{\mathrm{ES}}$ could be ignored. An exception to this analysis might occur if the active site were next to a paramagnetic centre, such as is found in metalloproteins for which $T_{1}^{\mathrm{ES}}$ could be very much shorter than predicted (see the relaxation theory section above).

### 5.3 Enzyme inhibition and hyperpolarized substrate kinetics

Our formalism can be readily extended to account for the influence of a ligand/solute to inhibit an enzyme. The simplest case is when a solute binds reversibly to the free enzyme E to form an enzyme–inhibitor complex EI ; hence, the enzyme becomes unable to bind and react with its substrate S . To describe this scenario, Eq. (68) is modified to include an additional pathway for the loss of free enzyme:

76 $$\begin{array}{rl} \frac{d\left[E\left(t\right)\right]}{dt} & =-k_{1}\left[E\left(t\right)\right]\left[S^{*}\left(t\right)+S\left(t\right)\right]\\ & +\left(k_{-1}+k_{2}\right)\left[{\mathrm{ES}}^{*}\left(t\right)+\mathrm{ES}\left(t\right)\right]\\ & -k_{-2}\left[E\left(t\right)\right]\left[P^{*}\left(t\right)+P\left(t\right)\right]\\ & -k_{3}\left[E\left(t\right)\right]\left[I\left(t\right)\right]+k_{-3}\left[\mathrm{EI}\left(t\right)\right]. \end{array}$$

The model is now extended to include differential equations describing the concentration of the inhibitor $\left[I\left(t\right)\right]$ and the enzyme–inhibitor complex $\left[\mathrm{EI}\left(t\right)\right]:$

77 $$\begin{array}{rl} & \frac{d\left[I\left(t\right)\right]}{dt}=-k_{3}\left[E\left(t\right)\right]\left[I\left(t\right)\right]+k_{-3}\left[\mathrm{EI}\left(t\right)\right],\\ & \frac{d\left[\mathrm{EI}\left(t\right)\right]}{dt}=k_{3}\left[E\left(t\right)\right]\left[I\left(t\right)\right]-k_{-3}\left[\mathrm{EI}\left(t\right)\right]. \end{array}$$

Such equations can be incorporated into the Michaelis–Menten equations, and we develop this next.

#### 5.3.1 Types of enzyme inhibition

There are three commonly encountered types of reversible enzyme inhibition (Kuchel, 2009): (i) a *competitive* inhibitor is structurally similar to the substrate and binds preferentially in the active site of the free enzyme, E , thus preventing the substrate from binding and reacting; (ii) an *uncompetitive* inhibitor binds only to the enzyme–substrate complex and therefore causes substrate-concentration-dependent inhibition; and (iii), a *non-competitive* inhibitor binds to both the free enzyme and to the enzyme–substrate complex; it causes a conformational change at the active site that inhibits (or even enhances) the reaction. Such an effect is referred to as allosteric inhibition (or activation).

Accounting for all three scenarios, the free enzyme concentration is given by

78 $$\begin{array}{rl} \left[E(t)\right] & =[E{]}_{0}-[\mathrm{EI}(t)]-[{\mathrm{ES}}^{*}(t)+\mathrm{ES}(t)]\\ & -\left[{\mathrm{ESI}}^{*}\left(t\right)+\mathrm{ESI}\left(t\right)\right]. \end{array}$$

Substituting

79 $$\alpha =1+\frac{\left[I\left(t\right)\right]}{K_{I}}\;\mathrm{and}\;\alpha^{\prime }=1+\frac{\left[I\left(t\right)\right]}{K_{I}^{\prime }},$$

where $K_{I}=\left[E\left(t\right)\right]\left[I\left(t\right)\right]/\left[\mathrm{EI}\left(t\right)\right]$ and $K_{I}^{\prime }=\left[\mathrm{ES}\left(t\right)\right]\left[I\left(t\right)\right]/\left[\mathrm{ESI}\left(t\right)\right]$ , yields

80 $$\frac{d\left(\left[P^{*}\left(t\right)+P\left(t\right)\right]\right)}{dt}=\frac{k_{2}[E{]}_{0}[S^{*}\left(t\right)+S\left(t\right)]}{\alpha K_{M}+\alpha^{\prime }\left[S^{*}\left(t\right)+S\left(t\right)\right]}.$$

The three types of enzyme inhibition can be distinguished by their influence on the kinetic parameters that are estimated in specially designed experiments performed on the enzyme over a range of substrate and inhibitor concentrations (Kuchel, 2009): (i) competitive inhibitors cause an increase in apparent $K_{M}$ value, while $V_{\max}$ is unchanged; (ii) uncompetitive inhibitors cause a reduction in $V_{\max}$ , while the apparent $K_{M}$ is unchanged; and (iii) non-competitive inhibitors cause both a reduction in $V_{\max}$ and an increase in apparent $K_{M}$ .

An additional effect that can be considered is where either the substrate of the reaction $\left[S\left(t\right)\right]$ or the product of the reaction, $\left[P\left(t\right)\right]$ , acts as the inhibitor, called unsurprisingly “substrate inhibition” and “product inhibition”, respectively. The relevant enzyme kinetic equations are composed by substituting $\left[I\left(t\right)\right]=\left[S^{*}\left(t\right)+S\left(t\right)\right]$ or $\left[I\left(t\right)\right]=\left[P^{*}\left(t\right)+P\left(t\right)\right]$ in the above equations.

## 6 Cofactors and unlabelled pools – lactate dehydrogenase

**Figure 8:**
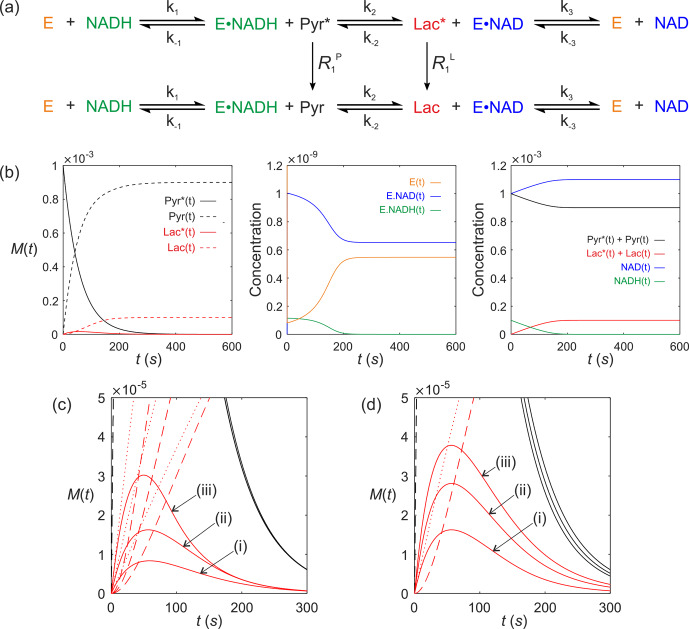
Simulated kinetics of LDH for exchange of solutes, $E+\mathrm{NADH}↔E\cdot \mathrm{NADH}+{\mathrm{Pyr}}^{*}↔E\cdot \mathrm{NAD}+{\mathrm{Lac}}^{*}↔E+{\mathrm{NAD}}^{+}$ , conforming to conservation of mass, assuming initial hyperpolarized magnetization of only solute ${\mathrm{Pyr}}^{*}\left(0\right)=0.001$ and ${\left[E\right]}_{0}=1.2\times 10^{-9}$ M. Longitudinal relaxation rate constants were $R_{1}^{P}=R_{1}^{L}=1/60$ s ${}^{-1}$ . Rate constants were $k_{1}=1.03\times 10^{8}$ M ${}^{-1}$ s ${}^{-1}$ , $k_{-1}=549$ s ${}^{-1}$ , $k_{2}=6.72\times 10^{6}$ M ${}^{-1}$ s ${}^{-1}$ , $k_{-2}=3.44\times 10^{4}$ M ${}^{-1}$ s ${}^{-1}$ , $k_{3}=842$ s ${}^{-1}$ and $k_{-3}=9.12\times 10^{5}$ M ${}^{-1}$ s ${}^{-1}$ . Initial cofactor concentrations were $\left[\mathrm{NADH}\left(0\right)\right]=1.0\times 10^{-4}$ M and $\left[\mathrm{NAD}\left(0\right)\right]=1.0\times 10^{-3}$ M. **(b)** Simulated time dependence ${\mathrm{Pyr}}^{*}\left(t\right)$ , $\mathrm{Pyr}\left(t\right)$ , ${\mathrm{Lac}}^{*}\left(t\right)$ and $\mathrm{Lac}\left(t\right)$ , left panel, [ E(t)] , [ E.NAD(t)] and [ E.NADH(t)] , middle panel, and ${\mathrm{Pyr}}^{*}\left(t\right)+\mathrm{Pyr}\left(t\right)=\left[\mathrm{Pyr}\left(t\right)\right]$ , ${\mathrm{Lac}}^{*}\left(t\right)+\mathrm{Lac}\left(t\right)=\left[\mathrm{Lac}\left(t\right)\right]$ , [ NAD(t)] and [ $\mathrm{NADH}\left(t\right)]$ , right panel. **(c)** Simulations of the time dependence of ${\mathrm{Lac}}^{*}\left(t\right)$ under the conditions that ${\left[E\right]}_{0}=$ (i) $0.6\times 10^{-9}$ M; (ii) $1.2\times 10^{-9}$ M; and (iii) $2.4\times 10^{-9}$ M, while all other parameters remained unchanged. **(d)** Simulations of the time dependence of ${\mathrm{Lac}}^{*}\left(t\right)$ under the conditions that $\mathrm{Lac}\left(0\right)=$ (i) 0 mM; (ii) 20 mM; and (iii) 40 mM, while all other parameters remained unchanged.

We now consider a real system that is of contemporary interest for in vivo clinical studies using dDNP. It is lactate dehydrogenase (LDH; E.C. 1.1.1.27). Consider the LDH-catalysed reaction of a hyperpolarized substrate; it follows an ordered sequential reaction in which $E+\mathrm{NADH}↔E\cdot \mathrm{NADH}+{\mathrm{Pyr}}^{*}↔E\cdot \mathrm{NAD}+{\mathrm{Lac}}^{*}↔E+{\mathrm{NAD}}^{+}$ . Again, we assume that relaxation of magnetization occurs through $T_{1}$ processes to form a pool of reactants $E+\mathrm{NADH}↔E\cdot \mathrm{NADH}+\mathrm{Pyr}↔E\cdot \mathrm{NAD}+\mathrm{Lac}↔E+{\mathrm{NAD}}^{+}$ as shown in Fig. 8a. The relevant differential equations used to describe the kinetics are (omitting the square brackets that denote concentration)

81dPyr∗tdt=-k2E.NADHtPyr∗t+k-2E.NADtLac∗t-R1PPyr∗t,82dLac∗tdt=k2E.NADHtPyr∗t-k-2E.NADtLac∗t-R1LLac∗t,83dPyrtdt=-k2E.NADHtPyrt+k-2E.NADtLact+R1PPyr∗t,84dLactdt=k2E.NADHtPyrt-k-2E.NADtLact+R1LLac∗t,85dNADHtdt=-k1EtNADHt+k-1E.NADHt,86dNADtdt=k3E.NADt-k-3EtNADt,87dE.NADHtdt=k1EtNADHt-k-1E.NADHt-k2E.NADHt(Pyr∗t+Pyr(t))+k-2E.NADt(Lac∗t+Lac(t)),88dE.NADtdt=k2E.NADHt(Pyr∗t+Pyr(t))-k-2E.NADt(Lac∗t+Lac(t))-k3E.NADt+k-3EtNADt,89dEtdt=-k1EtNADHt+k-1E.NADHt+k3E.NADt-k-3EtNADt,

where $E\left(t\right)$ is the concentration of free enzyme, $\mathrm{NAD}\left(t\right)$ and $\mathrm{NADH}\left(t\right)$ are the concentrations of the free cofactors, $E.\mathrm{NAD}\left(t\right)$ and $E.\mathrm{NADH}\left(t\right)$ are the concentrations of the enzyme–cofactor complexes and $\mathrm{Pyr}\left(t\right)$ and $\mathrm{Lac}\left(t\right)$ are the free substrates with relaxation rate constants $R_{1}^{P}$ and $R_{1}^{L}$ , respectively.

Mass is conserved, as is confirmed by the fact that $d\left({\mathrm{Pyr}}^{*}\left(t\right)+\mathrm{Pyr}\left(t\right)+{\mathrm{Lac}}^{*}\left(t\right)+\mathrm{Lac}\left(t\right)\right)/dt=0$ . Enzyme concentration is conserved, as is confirmed by $d\left(E.\mathrm{NADH}\left(t\right)+E.\mathrm{NAD}\left(t\right)+E\left(t\right)\right)/dt=0$ , and cofactor pools are conserved, as is confirmed by $d\left(\mathrm{NADH}\left(t\right)+\mathrm{NAD}\left(t\right)+E.\mathrm{NADH}\left(t\right)+E.\mathrm{NAD}\left(t\right)\right)/dt=0$ . Therefore, provided ${\mathrm{Pyr}}^{*}\left(0\right)+\mathrm{Pyr}\left(0\right)={\left[\mathrm{Pyr}\right]}_{0}$ and ${\mathrm{Lac}}^{*}\left(0\right)+\mathrm{Lac}\left(0\right)={\left[\mathrm{Lac}\right]}_{0}$ , then ${\mathrm{Pyr}}^{*}\left(t\right)+\mathrm{Pyr}\left(t\right)=\left[\mathrm{Pyr}\left(t\right)\right]$ and ${\mathrm{Lac}}^{*}\left(t\right)+\mathrm{Lac}\left(t\right)=\left[\mathrm{Lac}\left(t\right)\right]$ , respectively.

Equations (81)–(89) can be written in matrix vector form, given by Eq. (A90); see Appendix. We can apply a similarity transform, given by Eq. (A91) (see Appendix), to yield an equation of motion in the transformed basis vector given by Eq. (A92); see Appendix.

Figure 8b shows numerical simulations of the time evolution of the system that is described by Eqs. (81)–(89) with initial hyperpolarized signal/concentration (see above for a comment on this aspect) ${\mathrm{Pyr}}^{*}\left(t\right)=0.001$ and longitudinal relaxation rate constants $R_{1}^{P}=R_{1}^{L}=1/60$ s ${}^{-1}$ . The kinetic parameters used for lactate dehydrogenase were as previously published (Zewe and Fromm, 1962; Witney et al., 2011) for the rabbit muscle enzyme. Enzyme concentration was ${\left[E\right]}_{0}=1.2\times 10^{-9}$ M and rate constants were $k_{1}=1.03\times 10^{8}$ M ${}^{-1}$ s ${}^{-1}$ , $k_{-1}=549$ s ${}^{-1}$ , $k_{2}=6.72\times 10^{6}$ M ${}^{-1}$ s ${}^{-1}$ , $k_{-2}=3.44\times 10^{4}$ M ${}^{-1}$ s ${}^{-1}$ , $k_{3}=842$ s ${}^{-1}$ , and $k_{-3}=9.12\times 10^{5}$ M ${}^{-1}$ s ${}^{-1}$ .

The computed time dependence of polarized and unpolarized pools ${\mathrm{Pyr}}^{*}\left(t\right)$ , $\mathrm{Pyr}\left(t\right)$ , ${\mathrm{Lac}}^{*}\left(t\right)$ and $\mathrm{Lac}\left(t\right)$ are shown in Fig. 8b, left column of plots. The time dependences of [ E(t) ], [ E.NAD(t) ] and [ E.NADH(t) ] are shown in Fig. 8(b), middle panel. The time dependences of ${\mathrm{Pyr}}^{*}\left(t\right)+\mathrm{Pyr}\left(t\right)=\left[\mathrm{Pyr}\left(t\right)\right]$ , ${\mathrm{Lac}}^{*}\left(t\right)+\mathrm{Lac}\left(t\right)=\left[\mathrm{Lac}\left(t\right)\right]$ , [ NAD(t) ] and [ $\mathrm{NADH}\left(t\right)$ ] are shown in Fig. 8b, right panels. Several interesting features are evident. First, the model predicted the expected time dependences of both hyperpolarized pyruvate ${\mathrm{Pyr}}^{*}\left(t\right)$ and its conversion to ${\mathrm{Lac}}^{*}\left(t\right)$ . Under the conditions of the simulation, the free enzyme [ E(t) ] was rapidly depleted to form an equilibrium of [ E.NAD(t) ] and [ E.NADH(t) ]. During the reaction with ${\mathrm{Pyr}}^{*}\left(t\right)$ , the equilibrium position of the reaction was altered to give a final equilibrium position that could then be appreciated from the total pools of ${\mathrm{Pyr}}^{*}\left(t\right)+\mathrm{Pyr}\left(t\right)=\left[\mathrm{Pyr}\left(t\right)\right]$ and ${\mathrm{Lac}}^{*}\left(t\right)+\mathrm{Lac}\left(t\right)=\left[\mathrm{Lac}\left(t\right)\right]$ , which predicts a net conversion of $\left[\mathrm{Pyr}\left(t\right)\right]$ to $\left[\mathrm{Lac}\left(t\right)\right]$ of ∼10 %. Also note, from this simulation, that the activity of LDH switches off at t=200 s since the concentration of [ NADH(t) ] is limiting in this simulation; i.e. it becomes depleted. This does not happen if [ NADH(t) ] is increased. In a normal cellular context NADH would be regenerated by glyceraldehyde-3-phosphate dehydrogenase during glycolysis.

Finally, we consider real case scenarios that are reported in the literature, i.e. measurement of hyperpolarized [1- ${}^{13}$ C] pyruvate kinetics in living cells (Andersson et al., 2007; Day et al., 2007; Karlsson et al., 2007; Hill et al., 2013a, b; Lin et al., 2014; Pagès et al., 2014; Beloueche-Babari et al., 2017). Figure 8c shows the situation where the LDH expression level is altered, e.g. by the progression of disease (LDH expression is known to be upregulated in more aggressive cancer phenotypes – Albers et al., 2008) or downregulation during therapy (Ward et al., 2010), which can be explored through the value of ${\left[E\right]}_{0}$ . Figure 8c shows simulations of the ${\mathrm{Lac}}^{*}\left(t\right)$ signal under the conditions that ${\left[E\right]}_{0}=$ (i) $0.6\times 10^{-9}$ M, (ii) $1.2\times 10^{-9}$ M, and (iii) $2.4\times 10^{-9}$ M, while all other parameters remained unchanged, relative to those used for Fig. 8b. It is apparent that increased enzyme expression leads to an increase in the apparent rate of conversion of ${\mathrm{Pyr}}^{*}\left(t\right)$ to ${\mathrm{Lac}}^{*}\left(t\right)$ even in the absence of a change in enzyme activity, as seen in real experiments. Another situation that is frequently encountered is the change in the pool size of endogenous lactate, e.g. in response to hypoxia, which can be explored through the parameter $\mathrm{Lac}\left(0\right)$ . Figure 8d shows simulations of the ${\mathrm{Lac}}^{*}\left(t\right)$ signal under the conditions that $\mathrm{Lac}\left(0\right)=$ (i) 0 mM, (ii) 20 mM, and (iii) 40 mM, while all other parameters remained unchanged relative to those used to generate Fig. 8b. The model therefore predicts that an increased pool of endogenous unpolarized lactate leads to an increase in the rate of conversion of ${\mathrm{Pyr}}^{*}\left(t\right)$ to ${\mathrm{Lac}}^{*}\left(t\right)$ , as reported in the literature (Day et al., 2007).

## 7 Conclusions

We have described an approach to formulating the kinetic master equations that describe the time evolution of hyperpolarized ${}^{13}$ C NMR signals in reacting (bio)chemical systems, including enzymes with two or more substrates, and various enzyme reaction mechanisms as classified by Cleland. The modelling can be the basis for simulating many pertinent features that are seen in dDNP experiments. Derivation of the Michaelis–Menten equation in the context of dDNP experiments illustrates why formation of a hyperpolarized enzyme–substrate complex does *not* (typically) cause an appreciable loss of the signal from the substrate or product. It was also able to answer why the concentration of an unlabelled pool of substrate, for example ${}^{12}$ C lactate, causes an increase in the rate of exchange of the ${}^{13}$ C-labelled pool and to what extent the equilibrium position of an enzyme-catalysed reaction, for example LDH, is altered upon adding hyperpolarized substrate. The formalism described here should contribute to a fuller mechanistic understanding of the time courses derived from dDNP experiments and will be relevant to ongoing clinical applications using dDNP.

## Supplement

10.5194/mr-2-421-2021-supplement The supplement related to this article is available online at: https://doi.org/10.5194/mr-2-421-2021-supplement.

## Data Availability

All Matlab codes are reproduced in the Supplement and are free to adapt for personal use. For any further clarification please contact the corresponding author directly.

## Appendix

The supplement related to this article is available online at: https://doi.org/10.5194/mr-2-421-2021-supplement.
