# Supplementary material for: Extended Bloch–McConnell equations for mechanistic analysis of hyperpolarized 13C magnetic resonance experiments on enzyme systems
Source: Magn Reson (Gott). 2021 Jun 15;2(1):421–46. doi: 10.5194/mr-2-421-2021 (PMC10539799; doi:10.5194/mr-2-421-2021)
Supplement: The supplement related to this article is available online at: https://doi.org/10.5194/mr-2-421-2021-supplement. [file mr-2-421-supplement.pdf]

Supplement of Magn. Reson., 2, 421–446, 2021
https://doi.org/10.5194/mr-2-421-2021-supplement
© Author(s) 2021. CC BY 4.0 License.

Supplement of

Extended Bloch–McConnell equations for mechanistic analysis of hyperpolarized ^{13}C magnetic resonance experiments on enzyme systems

Thomas R. Eykyn et al.

Correspondence to: Thomas R. Eykyn (thomas.eykyn@kcl.ac.uk)

The copyright of individual parts of the supplement might differ from the article licence.

Supplemental Information - Extended Bloch-McConnell equations for mechanistic analysis of hyperpolarized ^{13}C magnetic resonance experiments on enzyme systems

Matlab scripts used for simulation in Figures 1-8. These are provided here in full to enable a more detailed exploration of influence of different parameters on the simulations. Readers are invited to contact the corresponding author should further clarification be required.

S1 Matlab program for numerically solving the Bloch McConnell equations for Figure 1

```
%%
% Set equilibrium magnetizations Meq

    MeqA = 1; MeqB = 0.8;

%%
% Set initial magnetization M0 in basis vector [Ax Ay Az Bx By Bz]

    M0 = [0,0,MeqA,0,0,MeqB];

%%
% Set relaxation rate constants r1 and r2, exchange rate constants k and
% peak frequencies freq

    r1 = 1; r2 = 1;
    kAB = 0; kBA = 0;
    freq_A = 10*2*pi; freq_B = -10*2*pi;

%%
% Set spectral acquisition parameters sw = sweep width, td = # time domain
% points, dt = dwell time

    sw = 60; td = 256; dt = 1/sw;

%%
% Set RF pulse parameters, B1 = field strength, phi = RF phase offset =
% RF offset, t_pulse = RF pulse duration

    B1 = 1500*2*pi; phi = 90*pi/180; offset = 0*2*pi;
    flip_angle = 90; t_pulse = flip_angle*pi/(180*B1);

%%
% Set input parameters as vector xIn

    xIn  = [r1,r2,freq_A,freq_B,B1,offset,phi,MeqA,MeqB,kAB,kBA];

%%
% Calculate evolution during pulse using DEs BlochMcConnell

    options = odeset('RelTol',1e-6,'AbsTol',1e-6);
    [~,Mt] = ode45(@BlochMcConnell,[0,t_pulse],M0,options,xIn);
    B1 = 0; xIn(5) = B1;

%%
% Calculate evolution during FID using DEs BlochMcConnell

    options = odeset('RelTol',1e-6,'AbsTol',1e-6);
    [t,Mt] = ode45(@BlochMcConnell,dt:dt:td*dt,Mt(end,:),options,xIn);
```

```matlab
%%
% Define observable magnetization Ax - i*Ay + Bx - i*By;

    Obs = Mt(:,1) - 1i*Mt(:,2) + Mt(:,4) - 1i*Mt(:,5);

%%
% Calculate fid and spectrum

    noise = randn(1,td)' + 1i*randn(1,td)';
    lb = 0; phase = 0;
    fid = (Obs + noise/10).*exp(-lb*t)*exp(1i*phase*pi/180);
    sig = fft(fid);
    spec = [sig(td/2+1:td);sig(1:td/2)];
    freq_scale = sw/2:-sw/(td-1):-sw/2;

%%
% Plot results

    subplot(1,3,1); plot(t,real(fid),'k');
    subplot(1,3,2); plot(freq_scale,real(spec),'k');
    subplot(1,3,3); plot(t,Mt(:,3),'k',t,Mt(:,6),'r');

%%
function dMt = BlochMcConnell(t,Mt,xIn)

    dMt = zeros(6,1);

    r1 = xIn(1);      r2 = xIn(2);
    freq_A = xIn(3); freq_B = xIn(4);
    MeqA = xIn(8);   MeqB = xIn(9);
    kAB = xIn(10);   kBA = xIn(11);

    wx = RFx(t,xIn(5),xIn(6),xIn(7));                    % Calculate pulse wx
    wy = RFy(t,xIn(5),xIn(6),xIn(7));                    % Calculate pulse wy

    dMt(1) = -(r2+kAB)*Mt(1) - freq_A*Mt(2) + wy*Mt(3) + kBA*Mt(4);       %Ax
    dMt(2) =  freq_A*Mt(1) - (r2+kAB)*Mt(2) - wx*Mt(3) + kBA*Mt(5);       %Ay
    dMt(3) = -wy*Mt(1) + wx*Mt(2) - (r1+kAB)*Mt(3) + kBA*Mt(6) + r1*MeqA; %Az
    dMt(4) =  kAB*Mt(1) - (r2+kBA)*Mt(4) - freq_B*Mt(5) + wy*Mt(6);       %Bx
    dMt(5) =  kAB*Mt(2) + freq_B*Mt(4) - (r2+kBA)*Mt(5) - wx*Mt(6);       %By
    dMt(6) =  kAB*Mt(3) - wy*Mt(4) + wx*Mt(5) - (r1+kBA)*Mt(6) + r1*MeqB; %Bz

    function [a] = RFx(tH,B1,offset,phi)
        a = B1*cos(offset*tH + phi);
    end

    function [a] = RFy(tH,B1,offset,phi)
        a = B1*sin(offset*tH + phi);
    end
end
```

S2 Matlab program for numerically solving the Bloch McConnell equations for hyperpolarized kinetics in Figure 2

```
%%
% Set equilibrium magnetizations Meq

    MeqA = 1; MeqB = 0.8;

%%
% Set initial magnetization M0 in basis vector [Ax Ay Az Bx By Bz]

    enhancement_factor = 1e4;
    M0 = [0,0,MeqA*enhancement_factor,0,0,0];

%%
% Set relaxation rate constants r1 and r2, exchange rate constants k and
% peak frequencies freq

    r1 = 1/60; r2 = 1;
    kAB = 0.005; kBA = 0.005;
    freq_A = 10*2*pi; freq_B = -10*2*pi;

%%
% Set spectral acquisition parameters sw = sweep width, td = # time domain
% points, dt = dwell time

    sw = 60; td = 256; dt = 1/sw;

%%
% Set RF pulse parameters, B1 = field strength, phi = RF phase, offset =
% RF offset, t_pulse = RF pulse duration

    B1 = 1500*2*pi; phi = 90*pi/180; offset = 0*2*pi;
    flip_angle = 1;

%%
% Set input parameters as vector xIn

    xIn  = [r1,r2,freq_A,freq_B,B1,offset,phi,MeqA,MeqB,kAB,kBA];

%%
% Create 2D data matrix SPEC and FID and time vector T, temporal resolution
% dt1

    td1 = 64; dt1 = 4.25; T = dt1:dt1:td1*dt1;
    SPEC=zeros(td,td1); FID=zeros(td,td1);
    Mt = M0;

%%
%

for m = 1:1:td1

    % Calculate evolution during pulse using DEs BlochMcConnell
    B1 = 1500*2*pi; xIn(5) = B1; t_pulse = flip_angle*pi/(180*B1);
    options = odeset('RelTol',1e-6,'AbsTol',1e-6);
    [~,Mt] = ode45(@BlochMcConnell,[0,t_pulse],Mt(end,:),options,xIn);
    B1 = 0;  xIn(5) = B1;

    % Calculate evolution during FID using DEs BlochMcConnell
    options = odeset('RelTol',1e-6,'AbsTol',1e-6);
    [t,Mt] = ode45(@BlochMcConnell,dt:dt:td*dt,Mt(end,:),options,xIn);

    % Calculate observable magnetization Ax - i*Ay + Bx - i*By;
    Obs = Mt(:,1) - 1i*Mt(:,2) + Mt(:,4) - 1i*Mt(:,5);
```

```
    % Calculate fid and spectrum
    noise = randn(1,td)' + 1i*randn(1,td)';
    lb = 0; phase = 0;
    fid = (Obs+noise./10).*exp(-lb*t).*exp(1i*phase*pi/180);
    sig = fft(fid);
    spec = [sig(td/2+1:td);sig(1:td/2)];
    freq_scale = sw/2:-sw/(td-1):-sw/2;

    FID(:,m)  = fid;
    SPEC(:,m) = spec;

end

%%
% % Plot results as a waterfall plot

   waterfall(freq_scale,T,real(SPEC'));axis square;axis tight;

%%
% % Plot 1st and last spectrum in time series

   figure;
   subplot(2,2,1); plot(t,real(FID(:,1)),'k');
   subplot(2,2,2); plot(freq_scale,real(SPEC(:,1)),'k');
   subplot(2,2,3); plot(t,real(FID(:,td1)),'k');
   subplot(2,2,4); plot(freq_scale,real(SPEC(:,td1)),'k');

%%
function dMt = BlochMcConnell(t,Mt,xIn)

    dMt = zeros(6,1);

    r1     = xIn(1);  r2     = xIn(2);
    freq_A = xIn(3);  freq_B = xIn(4);
    MeqA   = xIn(8);  MeqB   = xIn(9);
    kAB    = xIn(10); kBA    = xIn(11);

    wx = RFx(t,xIn(5),xIn(6),xIn(7));                    % Calculate pulse wx
    wy = RFy(t,xIn(5),xIn(6),xIn(7));                    % Calculate pulse wy

    dMt(1) = -(r2+kAB)*Mt(1) - freq_A*Mt(2) + wy*Mt(3) + kBA*Mt(4);       %Ax
    dMt(2) =  freq_A*Mt(1) - (r2+kAB)*Mt(2) - wx*Mt(3) + kBA*Mt(5);       %Ay
    dMt(3) = -wy*Mt(1) + wx*Mt(2) - (r1+kAB)*Mt(3) + kBA*Mt(6) + r1*MeqA; %Az
    dMt(4) =  kAB*Mt(1) - (r2+kBA)*Mt(4) - freq_B*Mt(5) + wy*Mt(6);       %Bx
    dMt(5) =  kAB*Mt(2) + freq_B*Mt(4) - (r2+kBA)*Mt(5) - wx*Mt(6);       %By
    dMt(6) =  kAB*Mt(3) - wy*Mt(4) + wx*Mt(5) - (r1+kBA)*Mt(6) + r1*MeqB; %Bz

    function [a] = RFx(tH,B1,offset,phi)
        a = B1*cos(offset*tH + phi);
    end

    function [a] = RFy(tH,B1,offset,phi)
        a = B1*sin(offset*tH + phi);
    end

end
```

S3 Matlab program for numerically calculating ^{13}C relaxation times based on a simple dipole-dipole plus chemical shift anisotropy mechanism in Figure 3

```
%%
% Set gyromagnetic ratios gamma of 1H and 13C

    gamma_H = 26.7522e7; gamma_C = 6.7283e7;              % rad s-1 T-1
%%
% Set chemical shift anisotropies csa of 1H and 13C

    csa_H = 10e-6; csa_C = -98e-6;

%%
% Set Planck's constant h, Boltzmann constant kB, vacuum permeability no

    h = 6.626176e-34; hbar = h/(2*pi); kB = 1.380658e-23; mu = 4*pi*1e-7;

%%
% Set vectors for magnetic field B0 and rotational correlation time tc

    B0 = (0.5:0.5:14.1)';
    x = -12:0.1:-7;
    tc = 10.^x;

%%
% Set Larmor frequencies

    wH = -gamma_H*B0;
    wC = -gamma_C*B0;

%%
% Set CH bond length, dipolar coupling constant and csa constant
%
% Set rch ~ 1.09e-10 one bond C-H distance

    rch = 1.45e-10;
    d = (mu/(4*pi))*(gamma_H*gamma_C*hbar/rch^3);
    c = gamma_C*B0*csa_C;

%%
% Calculate spectral density functions

    J0 = 2*tc.*ones(length(B0),1);
    Jc = 2*tc./(1 + (wC.^2*tc.^2));
    Jh = 2*tc./(1 + (wH.^2*tc.^2));
    Jmin = 2*tc./(1 + ((wH - wC).^2*tc.^2));
    Jplus = 2*tc./(1 + ((wH + wC).^2*tc.^2));
    Jhh = 2*tc./(1 + ((wH + wH).^2*tc.^2));

%%
% Calculate dipole-dipole relaxation rate constants and time constants

    R1DD = d^2*((3/20)*Jc + (1/20)*Jmin + (3/10)*Jplus);
    R2DD = d^2*((1/10)*J0 + (3/40)*Jc + (1/40)*Jmin + (3/20)*Jh + (3/20)*Jplus);
    T1DD = 1./R1DD; T2DD = 1./R2DD;

%%
% Calculate CSA relaxation rate constants and time constants

    R1CSA = c.^2.*((1/15)*Jc);
    R2CSA = c.^2.*((2/45)*J0 + (1/30)*Jc);
    T1CSA = 1./R1CSA; T2CSA = 1./R2CSA;

%%
% Calculate sum of DD and CSA contributions to R1 and R2
```

```
    R1 = R1DD + R1CSA;
    R2 = R2DD + R2CSA;
    T1 = 1./R1; T2 = 1./R2;

%%
% Surface plot of T1 and T2 as a function of B0 and tauc

    subplot(1,2,1);
    mesh(B0,x,T1');axis square;
    subplot(1,2,2);
    mesh(B0,x,T2');axis square;
```

S4 Matlab program for numerically calculating hyperpolarized kinetics of a first order two-site exchange reaction A ↔ B in Figure 4

```
%%
% Set initial magnetizations A*(0) = A0, A(0) = a0, B*(0) = 0; B(0) = b0;

    A0 = 1; B0 = 0; a0 = 0; b0 = 0;

%%
% Set relaxation rate constants r1 and r2, exchange rate constants k

    r1A = 1/60; r1B = 1/60;
    kAB = 0.01; kBA = 0.005;

%%
% Set initial magnetization basis vector [A* B* A B]

    M0 = [A0,B0,a0,b0];

%%
% Set input parameters as vector xIn

    xIn  = [r1A,r1B,kAB,kBA];

%%
% Set time vector for simulation

    dt = 0.01; tmax = 600;
    t = 0:dt:tmax;

%%
% Calculate time evolution using DE function diff_eqs

    options = odeset('RelTol',1e-6,'AbsTol',1e-6);
    [tx,y] = ode15s(@diff_eqs,t,M0,options,xIn);

%%
% Plot results

    subplot(1,2,1);
    plot(t,y(:,1),'k',t,y(:,2),'r',t,y(:,3),'k--',t,y(:,4),'r--');
    subplot(1,2,2);
    plot(t,y(:,1)+y(:,3),'k',t,y(:,2)+y(:,4),'r');

%%
function dy = diff_eqs(~,y,xIn)

    dy = zeros(4,1);

    r1A = xIn(1); r1B = xIn(2);
    kAB = xIn(3); kBA = xIn(4);

    dy(1) = -kAB*y(1) + kBA*y(2) - r1A*y(1);               % A*
    dy(2) =  kAB*y(1) - kBA*y(2) - r1B*y(2);               % B*

    dy(3) = -kAB*y(3) + kBA*y(4) + r1A*y(1);               % A
    dy(4) =  kAB*y(3) - kBA*y(4) + r1B*y(2);               % B

end
```

S5 Matlab program for numerically calculating hyperpolarized kinetics of a first order three-site exchange reaction A ↔ B ↔ C in Figure 5

```matlab
%%
% Set initial magnetizations A*(0) = A0, B*(0) = B0; C*(0) = C0; A(0) =
% a(0); B(0) = b0; C(0) = c0;

    A0 = 1; B0 = 0; C0 = 0; a0 = 0; b0 = 0; c0 = 0;

%%
% Set relaxation rate constants r1 and r2, exchange rate constants k

    r1A = 1/60; r1B = 1/60;  r1C = 1/60;
    kAB = 0.01; kBA = 0.005; kBC = 0.01; kCB = 0.005;

%%
% Set initial magnetization basis vector [A* B* C* A B C]

    M0 = [A0,B0,C0,a0,b0,c0];

%%
% Set input parameters as vector xIn

    xIn  = [r1A,r1B,r1C,kAB,kBA,kBC,kCB];

%%
% Set time vector for simulation

    dt = 0.01; tmax = 600;
    t = 0:dt:tmax;

%%
% Calculate time evolution using DE function diff_eqs

    options = odeset('RelTol',1e-6,'AbsTol',1e-6);
    [t,y] = ode15s(@diff_eqs,t,M0,options,xIn);

%%
% Plot results

    subplot(1,2,1)
    plot(t,y(:,1),'k',t,y(:,2),'b',t,y(:,3),'r'); hold on;
    plot(t,y(:,4),'k--',t,y(:,5),'b--',t,y(:,6),'r--');
    subplot(1,2,2)
    plot(t,y(:,1)+y(:,4),'k',t,y(:,2)+y(:,5),'b',t,y(:,3)+y(:,6),'r');

%%
function dy = diff_eqs(~,y,xIn)

        dy = zeros(6,1);

        r1A = xIn(1); r1B = xIn(2); r1C = xIn(3);
        kAB = xIn(4); kBA = xIn(5); kBC = xIn(6); kCB = xIn(7);

        dy(1) = -kAB*y(1) + kBA*y(2) - r1A*y(1);                          % A*
        dy(2) =  kAB*y(1) - kBA*y(2) - kBC*y(2) + kCB*y(3) - r1B*y(2);    % B*
        dy(3) =  kBC*y(2) - kCB*y(3) - r1C*y(3);                          % C*

        dy(4) = -kAB*y(4) + kBA*y(5) + r1A*y(1);                          % A
        dy(5) =  kAB*y(4) - kBA*y(5) - kBC*y(5) + kCB*y(6) + r1B*y(2);    % B
        dy(6) =  kBC*y(5) - kCB*y(6) + r1C*y(3);                          % C

end
```

S6 Matlab program for numerically calculating hyperpolarized kinetics of a second order two-site exchange reaction A + C ↔ B + D in Figure 6

```
%%
% Set initial magnetizations A*(0) = A0, B*(0) = 0; A(0) = a0, B(0) = b0;
% C(0) = c0; D(0) = d0;

    A0 = 1; B0 = 0; a0 = 0; b0 = 0; c0 = 0.95; d0 = 0.05;

%%
% Set relaxation rate constants r1 and r2, exchange rate constants k

    r1A = 1/60; r1B = 1/60;
    kAB = 0.01; kBA = 0.005;

%%
% Set initial magnetization M0 in basis vector [A* B* A B C D]

    M0 = [A0,B0,a0,b0,c0,d0];

%%
% Set input parameters as vector xIn

    xIn = [r1A,r1B,kAB,kBA];

%%
% Set time vector for simulation

    dt = 0.01; tmax = 600;
    t = 0:dt:tmax;

%%
% Calculate time evolution using DE function diff_eqs

    options = odeset('RelTol',1e-6,'AbsTol',1e-6);
    [t,y] = ode15s(@diff_eqs,t,M0,options,xIn);

%%
% Plot results

    subplot(1,2,1);
    plot(t,y(:,1),'k',t,y(:,2),'r',t,y(:,3),'k--',t,y(:,4),'r--');
    subplot(1,2,2);
    plot(t,y(:,1)+y(:,3),'k',t,y(:,2)+y(:,4),'r',t,y(:,5),'b',t,y(:,6),'g');

%%
function dy = diff_eqs(~,y,xIn)

        dy = zeros(6,1);

        r1A = xIn(1); r1B = xIn(2);
        kAB = xIn(3); kBA = xIn(4);

        dy(1) = -kAB*y(1)*y(5) + kBA*y(2)*y(6) - r1A*y(1);          % A*
        dy(2) =  kAB*y(1)*y(5) - kBA*y(2)*y(6) - r1B*y(2);          % B*

        dy(3) = -kAB*y(3)*y(5) + kBA*y(4)*y(6) + r1A*y(1);          % A
        dy(4) =  kAB*y(3)*y(5) - kBA*y(4)*y(6) + r1B*y(2);          % B

        dy(5) = -kAB*y(5)*(y(1) + y(3)) + kBA*y(6)*(y(2) + y(4));   % C
        dy(6) =  kAB*y(5)*(y(1) + y(3)) - kBA*y(6)*(y(2) + y(4));   % D
end
```

S7 Matlab program for numerically calculating Michaelis Menten kinetics of a hyperpolarized substrate in Figure 7

```
%%
% Set initial magnetizations S*(0) = S0, ES*(0) = ES0; P*(0) = P0, S(0)
% = s0; ES(0) = es0; P(0) = p0; E(0) = E0;

    S0 = 1e-3; ES0 = 0; P0 = 0; s0 = 0; es0 = 0; p0 = 0; E0 = 1e-9;
%%
% Set relaxation rate constants r1 and exchange rate constants k

    r1S = 1/60; r1ES = 1/60; r1P = 1/60;
    kplus1 = 1e7; kmin1 = 1e2; kplus2 = 5e3; kmin2 = 0;

%%
% Set initial magnetization M0 in basis vector [S* ES* P*  S  ES  P  E]

    M0 = [S0,ES0,P0,s0,es0,p0,E0];

%%
% Set input parameters as vector xIn

    xIn  = [r1S,r1ES,r1P,kplus1,kmin1,kplus2,kmin2];

%%
% Set time vector for simulation

    dt = 0.01; tmax = 600;
    t = 0:dt:tmax;
%%
% Calculate time evolution using DE function diff_eqs

    options = odeset('RelTol',1e-10,'AbsTol',1e-10);
    [t,y] = ode15s(@diff_eqs,t,M0,options,xIn);
%%
% Plot results

    subplot(2,2,1)
    plot(t,y(:,1),'k',t,y(:,4),'k--',t,y(:,3),'r',t,y(:,6),'r--');
    subplot(2,2,2)
    plot(t,y(:,1)+y(:,4),'k',t,y(:,3)+y(:,6),'r');
    subplot(2,2,3)
    plot(t,y(:,2),'g',t,y(:,5),'g--');
    subplot(2,2,4)
    plot(t,y(:,2)+y(:,5),'g',t,y(:,7),'y');

%%
function dy = diff_eqs(~,y,xIn)

    dy = zeros(7,1);

    r1S    = xIn(1); r1ES  = xIn(2); r1P = xIn(3);
    kplus1 = xIn(4); kmin1 = xIn(5);
    kplus2 = xIn(6); kmin2 = xIn(7);

    dy(1) = -kplus1*y(1)*y(7) + kmin1*y(2) - r1S*y(1);
    dy(2) =  kplus1*y(1)*y(7) - kmin1*y(2) - kplus2*y(2) + kmin2*y(3)*y(7) - r1ES*y(3);
    dy(3) =  kplus2*y(2) - kmin2*y(3)*y(7) - r1P*y(3);

    dy(4) = -kplus1*y(4)*y(7) + kmin1*y(5) + r1S*y(1);
    dy(5) =  kplus1*y(4)*y(7) - kmin1*y(5) - kplus2*y(5) + kmin2*y(6)*y(7) + r1ES*y(3);
    dy(6) =  kplus2*y(5) - kmin2*y(6)*y(7) + r1P*y(3);

    dy(7) = -kplus1*y(7)*(y(1)+y(4)) + (kmin1+kplus2)*(y(2)+y(5)) - kmin2*y(7)*(y(3)+y(6));

end
```

S8 Matlab program for numerically calculating Lactate dehydrogenase kinetics for hyperpolarized pyruvate in Figure 8

```
%%
% Set initial magnetizations Pyr*(0) = P0; Lac*(0) = L0; Pyr(0) = p0; Lac(0)
% = l0; NADH(0) = nadh0; NAD(0) = nad0; E.NADH(0) = Enadh0; E.NAD(0) = Enad0;
% E(0) = E0;

    P0 = 1e-3; L0 = 0; p0 = 0; l0 = 0; nadh0 = 0.1e-3; nad0 = 1e-3;
    Enadh0 = 0; Enad0 = 0; E0 = 1.2e-9;

%%
% Set relaxation rate constants r1 and exchange rate constants k

    r1P = 1/60;       r1L = 1/60;
    kplus1 = 1.03e8; kmin1 = 549;
    kplus2 = 6.72e6; kmin2 = 3.44e4;
    kplus3 = 842;    kmin3 = 9.12e5;

%%
% Set initial magnetization M0 in basis vector [P* L* P L NADH NAD E.NADH
% E.NAD E]

    M0 = [P0,L0,p0,l0,nadh0,nad0,Enadh0,Enad0,E0];

%%
% Set input parameters as vector xIn

    xIn  = [r1P,r1L,kplus1,kmin1,kplus2,kmin2,kplus3,kmin3];

%%
% Set time vector for simulation

    dt = 0.01; tmax = 600;
    t = 0:dt:tmax;

%%
% Calculate time evolution using DEs

    options = odeset('RelTol',1e-10,'AbsTol',1e-10);
    [t,y] = ode15s(@diff_eqs,t,M0,options,xIn);

%%
% Plot results

    subplot(1,3,1)
    plot(t,y(:,1),'k',t,y(:,3),'k--',t,y(:,2),'r',t,y(:,4),'r--');
    subplot(1,3,2)
    plot(t,y(:,7),'g',t,y(:,8),'b',t,y(:,9),'y');
    subplot(1,3,3)
    plot(t,y(:,1)+y(:,3),'k',t,y(:,2)+y(:,4),'r',t,y(:,5),'g',t,y(:,6),'b');

%%
function dy = diff_eqs(~,y,xIn)

    dy = zeros(9,1);

    r1P    = xIn(1); r1L    = xIn(2);
    kplus1 = xIn(3); kmin1 = xIn(4);
    kplus2 = xIn(5); kmin2 = xIn(6);
    kplus3 = xIn(7); kmin3 = xIn(8);

    dy(1) = -kplus2*y(1)*y(7) + kmin2*y(2)*y(8) - r1P*y(1);
    dy(2) =  kplus2*y(1)*y(7) - kmin2*y(2)*y(8) - r1L*y(2);
```

```
    dy(3) = -kplus2*y(3)*y(7) + kmin2*y(4)*y(8) + r1P*y(1);
    dy(4) =  kplus2*y(3)*y(7) - kmin2*y(4)*y(8) + r1L*y(2);

    dy(5) = -kplus1*y(5)*y(9) + kmin1*y(7);
    dy(6) = -kmin3*y(6)*y(9) + kplus3*y(8);

    dy(7) =  kplus1*y(5)*y(9) - kmin1*y(7) - kplus2*y(7)*(y(1)+y(3)) + kmin2*y(8)*(y(2)+y(4));
    dy(8) =  kplus2*y(7)*(y(1)+y(3)) - kmin2*y(8)*(y(2)+y(4)) - kplus3*y(8) + kmin3*y(6)*y(9);

    dy(9) = -kplus1*y(5)*y(9) + kmin1*y(7) + kplus3*y(8) - kmin3*y(6)*y(9);

end
```
